# Multi-Epitope Vaccine for Monkeypox Using Pan-Genome and Reverse Vaccinology Approaches

**DOI:** 10.3390/v14112504

**Published:** 2022-11-12

**Authors:** Rayapadi G. Swetha, Soumya Basu, Sudha Ramaiah, Anand Anbarasu

**Affiliations:** 1 Medical and Biological Computing Laboratory, School of Biosciences and Technology (SBST), Vellore Institute of Technology (VIT), Vellore 632014, India; 2 Department of Biotechnology, School of Biosciences and Technology (SBST), Vellore Institute of Technology (VIT), Vellore 632014, India; 3 Department of Biosciences, School of Biosciences and Technology (SBST), Vellore Institute of Technology (VIT), Vellore 632014, India; 4 Department of Bio-Medical Sciences, School of Biosciences and Technology (SBST), Vellore Institute of Technology (VIT), Vellore 632014, India

**Keywords:** monkeypox, vaccine, epitope, pan-genome, immunoinformatics, virus

## Abstract

Outbreaks of monkeypox virus infections have imposed major health concerns worldwide, with high morbidity threats to children and immunocompromised adults. Although repurposed drugs and vaccines are being used to curb the disease, the evolving traits of the virus, exhibiting considerable genetic dynamicity, challenge the limits of a targeted treatment. A pan-genome-based reverse vaccinology approach can provide fast and efficient solutions to resolve persistent inconveniences in experimental vaccine design during an outbreak-exigency. The approach encompassed screening of available monkeypox whole genomes (n = 910) to identify viral targets. From 102 screened viral targets, viral proteins L5L, A28, and L5 were finalized based on their location, solubility, and antigenicity. The potential T-cell and B-cell epitopes were extracted from the proteins using immunoinformatics tools and algorithms. Multiple vaccine constructs were designed by combining the epitopes. Based on immunological properties, chemical stability, and structural quality, a novel multi-epitopic vaccine construct, V4, was finalized. Flexible-docking and coarse-dynamics simulation portrayed that the V4 had high binding affinity towards human HLA-proteins (binding energy < −15.0 kcal/mol) with low conformational fluctuations (<1 Å). Thus, the vaccine construct (V4) may act as an efficient vaccine to induce immunity against monkeypox, which encourages experimental validation and similar approaches against emerging viral infections.

## 1. Introduction

The global burden of monkeypox cases as a result of the 2022 outbreak is estimated to be ~73,500 (6.6% increase in October 2022) across 109 countries around the world, imposing high to moderate threats in most parts of the world [1]. Although the disease was initially endemic to the Democratic Republic of Congo (DRC) and parts of central Africa, with a case-fatality rate of 8–10%, population migrations have resulted in its emergence and re-emergence in Europe, America, and parts of the western Pacific region in the last two decades [2].

The monkeypox virus (MPXV), a double-stranded DNA (dsDNA) virus of the Orthopoxvirus genus, Chordopoxvirinae subfamily and Poxviridae family, is the etiological agent of monkeypox, a zoonotic illness having high morbidity threats in children and immunocompromised people [3]. Historical evidence suggests that smallpox vaccination engendered about 85% protection against monkeypox. However, the eradication of smallpox and subsequent discontinuation of the orthopoxvirus vaccine program for more than 40 years can be a major reason of the re-emergence of MPXV [3,4]. Another major cause of the resurgence of monkeypox is its genetic evolution; for example, four different lineages of the virus were identified from region-specific samples in DRC [2]. A recent study overviewed the epidemiology and evolution of MPXV in Nigeria and around the world, stating recombination events, and gene loss/gain in MPXV progression [4]. Although cross-protection through vaccinia-viral vaccines (JYNNEOSTM and ACAM2000) has been reported, there is no specific vaccine for MPXV to date [5,6]. The recommended high-dose antiviral drugs (tecovirimat, brincidofovir, cidofovir) for severe MPXV infections tend to impose hepatic malfunctions, whereas low-dose medications potentiate the risks of relapse [5,6,7]. The major drawback in the available vaccines against MPXV is the specificity and potential to elicit a long-lasting immune response, especially in the context of the evolving pathogen [8,9]. Amidst limited therapeutic regimens against MPXV, genome-wide analyses revealed the emerging clades of MPXV having the potency to cause havoc since they are highly contagious [2,4]. Hence, state-of-the-art solutions are imperative to combat this re-emerging pathogen. One of the best methods in the pipeline of drug development is the identification of new vaccination targets. Due to the availability of whole-genome sequence data, cross-disciplinary techniques can be used to identify viral targets for therapeutic intervention [10]. Traditional methods for vaccine design and drug identification are both inconvenient during a disease-outbreak in terms of time and economy. Consequently, genome-based technology has become a realistic option for identifying novel therapeutic targets and promiscuous multi-epitope vaccines [11,12,13]. Genome-based reverse vaccinology is a trusted computational technique for fast and efficient vaccine designs [14]. In this context, it can be mentioned that our research group has been working on genomics, structural bioinformatics and immunoinformatics to elucidate therapeutic solutions against emerging pathogenic entities [15,16,17,18,19,20,21]. In the present study, the reverse vaccinology approach was used to develop a multi-epitope vaccine to entice human immunity towards MPXV. The methodology entails creating multiple fragments (epitopes) from suitable MPXV-proteins to stimulate cellular and humoral immunity reactions, without concurrent health hazards. The epitopes were stacked in a suitable vaccine construct and its efficacy was checked through physicochemical, immunological, and structural properties. The study provides efficient leads for experimental vaccine design against MPXV.

## 2. Materials and Methods

### 2.1. Retrieval of Complete Genomes

In total, we have retrieved 2798 genomes of MPXV from the NCBI database. The genomes that were flagged as complete were screened and subsequently we manually curated those datasets to exclude duplicates. The genomes of MPXV with complete assembly (n = 910) were retrieved and downloaded in FASTA format (https://www.ncbi.nlm.nih.gov/labs/virus (accessed on 20 September 2022)). 

### 2.2. Genome Annotation and Pan-Genome Inference

All complete genomes (n = 910) of MPXV were reannotated de novo with Prokka v1.14.6 (https://github.com/tseemann/prokka (accessed on 20 September 2022)) [22]. Prokka is a genome annotation tool by the Pathogen Informatics team at the Wellcome Trust Sanger Institute that uses prodigal [23] for gene prediction. A pan-genome of MPXV was constructed by Roary v3.13.0 (https://github.com/sanger-pathogens/Roary (accessed on 21 September 2022 )) [24] with a 100% BLAST v2.9 identity threshold using the MAFFT multiple sequence alignment program v7.453 [25]. The annotated genomes obtained from Prokka were provided to Roary as an input. Roary used PRANK v.170427 [26] to generate the gene presence/absence matrix, a multi-FASTA alignment of core genes. 

### 2.3. Target Protein Prediction

An intersection operation was performed to obtain the core genes in all the subjected genomes. Then, the non-homologous sequences to the human host proteome were filtered out using BLASTp with an E-value > 0.005 [27]. This similarity search can avoid cross- reaction or adverse side effects against humans. The resultant non-homologous sequences were evaluated for subcellular localization using a new fusion classifier, Virus-mPLoc (http://csbio.sjtu.edu.cn/bioinf/virus-multi/ (accessed on 23 September 2022)). The knowledge of subcellular localization of viral proteins plays an important role in designing antiviral drugs [28]. The viral proteins located in the outer membrane were taken into account as target proteins in predicting vaccine constructs. Then, the predicted outer membrane proteins (OMPs) were subjected to check for their antigenicity and solubility. The antigenicity of filtered proteins was predicted using the VaxiJen v2.0 server (http://www.ddg-pharmfac.net/vaxijen/ (accessed on 23 September 2022)) and ANTIGENpro (http://scratch.proteomics.ics.uci.edu/ (accessed on 23 September 2022)). The VaxiJen server classifies a protein as an antigen based on auto cross-covariance transformation of protein sequences into uniform vectors of principal amino acid properties [29], whereas ANTIGENpro utilizes protein antigenicity microarray data to predict protein antigenicity [30]. Both these antigenicity predictive methods are sequence-based alignment-free predictors of protective antigens. SolPro (http://scratch.proteomics.ics.uci.edu/ (accessed on 23 September 2022)), a sequence- based prediction method, was used to identify the propensity of a protein to be soluble upon overexpression [31]. The proteins exhibiting antigenicity in both VaxiJen v2.0 server and ANTIGENpro and solubility were considered effective proteins for the construction of an epitope-based vaccine.

### 2.4. Epitope Prediction

The shortlisted proteins were submitted to multiple tools/servers to predict the potential MHC class-I epitopes, MHC class-II T-cell epitopes and B-cell epitopes, which induce good immune responses after binding [32].

#### 2.4.1. MHC Class-I T-Cell Epitope Prediction

The MHC class-I binding regions in the antigenic proteins were predicted by using three different platforms: ProPred1 (http://crdd.osdd.net/raghava/propred1/ (accessed on 26 September 2022)), MHC-I binding predictions (http://tools.iedb.org/mhci/ (accessed on 26 September 2022)) and class-I immunogenicity (http://tools.iedb.org/immunogenicity/ (accessed on 26 September 2022)). The latter two are from the Immune Database (IEDB) analysis resource. The ProPred1 is a matrix-based web server, used to find the binding regions and proteasome cleavage sites in the given protein sequence for 47 MHC class-I alleles. The server utilizes matrices obtained from the BIMAS server to identify promiscuous MHC binders [33] that bind to many alleles. All the available alleles were chosen for maximum coverage, and a 3% threshold was maintained in the proteasome filter and immunoproteasome. The MHC-I binding predictions of IEDB gather data from various binding prediction approaches and the Artificial Neural Network (ANN), which is the best method, was chosen to predict 9-mer peptides against 27 different frequently occurring Human Leukocyte Antigen (HLA) alleles [34]. The peptides with IC50 values < 50 nM are considered to show high affinity towards HLA alleles and were selected. The 9-mer peptides found to be promising in these two platforms were shortlisted and checked for their immunogenicity by the class-I immunogenicity tool. In this tool, the amino acids’ properties and their positions are taken into account to measure the immunogenicity score of the given peptides [35]. The peptides with >0.15 immunogenicity score were considered immunogenic. In addition, the toxicity and antigenicity of these peptides were evaluated by ToxinPred and the VaxiJen v2.0 server, respectively. The ToxinPred tool identifies highly toxic regions in the given peptides, and the Support Vector Machine was selected as the prediction method [36] with a cut-off value of 0.5. Finally, the non-toxic and antigenic epitopes were selected for vaccine construction. 

#### 2.4.2. MHC Class-II T-Cell Epitope Prediction

The prediction of peptide binding to MHC class-II molecules was performed by IEDB MHC-II binding predictions (http://tools.iedb.org/mhcii/ (accessed on 26 September 2022)). The tool incorporates nine prediction methods: 1. IEDB recommended, 2. consensus method, 3. combinatorial library, 4. NN-align −2.3, 5. NN-align 2.2, 6. SMM-align, 7. Sturniolo, 8. NetMHCIIpan −3.1, and 9. NetMHCIIpan −3.2 [37]. The consensus prediction method was picked up as it is widely used, and the peptides with percentile rank < 0.2 and IC50 < 50 nM were considered strong binders against a complete HLA reference set. Then, these peptides were assessed for their allele-independent CD4 T-cell immunogenicity by IEDB CD4 T-cell immunogenicity prediction tool (http://tools.iedb.org/CD4episcore/ (accessed on 26 September 2022)). The IEDB recommended a combined method to predict the MHC class-II binding peptides, and this method combines the predictions from 7-allele and immunogenicity method. The peptides can be considered immunogenic when their percentile rank is <66 [38]. The strong MHC class-II binding immunogenic epitopes were evaluated for their toxicity and antigenicity by ToxinPred [36] and VaxiJen v2.0 [29] servers, respectively. The immunogenic and antigenic epitopes with strong binding affinity and non-toxic were only selected for vaccine construction. 

#### 2.4.3. MHC Class-I/II Cluster Analysis

The shortlisted MHC class-I/II epitopes were double-checked by clustering MHC-restricted alleles based on their binding specificity using MHCcluster v2.0 (https://services.healthtech.dtu.dk/service.php?MHCcluster-2.0 (accessed on 26 September 2022)) [39]. The server produces a heat map and graphical tree to define the functional relationship between the predicted epitopes and HLA alleles. This process will strengthen our epitope predictions. 

#### 2.4.4. Linear B-Cell Epitope Prediction

The epitopes must be recognized by B-lymphocytes to initiate humoral immunity, which can eliminate the foreign bodies by secreting antibodies against antigens exposed in humans. The linear B-cell epitopes were identified by employing two tools: BepiPred v2.0 and ABCPred. BepiPred v2.0 (https://services.healthtech.dtu.dk/service.php?BepiPred-2.0 (accessed on 27 September 2022)) uses a random forest algorithm trained on the epitopes annotated from antigen–antibody structures [40] whereas ABCPred (http://crdd.osdd.net/raghava/abcpred/ (accessed on 27 September 2022)) uses ANN method of static length patterns to predict B-cell epitope in an antigenic sequence [41]. The default parameters were set in both tools with a cut-off value of 0.5, and the epitopes commonly presented in both tools were selected for vaccine construction.

### 2.5. Epitope Selection and Designing Vaccine Construct

The selected T-cell and B-cell epitopes from the aforementioned servers were compared manually. The overlapping and unique epitopes were selected for vaccine construction. We considered different combinations of these epitopes to make a novel vaccine with high immunogenicity, less allergic and non-toxic. To make an efficient vaccine, we sequentially added appropriate adjuvants, linkers, His Tag (six histamines) and PADRE (Pan HLA-DR reactive epitope) sequences to the shortlisted epitopes [42]. Later, these different blends of vaccine constructs were assessed for physio-chemical parameters.

### 2.6. Assessment of Vaccine Constructs

The physio-chemical and functional properties of the vaccine constructs were evaluated by the Expasy ProtParam tool (https://web.expasy.org/protparam/ (accessed on 27 September 2022)). The tool gives information on a protein’s molecular weight, instability index and grand average of hydropathicity (GRAVY) [43]. Additionally, the Allergen v2.0 (https://ddg-pharmfac.net/AllergenFP/ (accessed on 27 September 2022)) and VaxiJen v2.0 [29] were employed in the designed vaccine constructs to assess their allergenicity and antigenicity properties. The Allergen is a novel alignment-independent descriptor-based fingerprint approach for identifying the allergenicity of proteins [44]. 

### 2.7. Modeling and Docking Methods

Based on the physicochemical and immunological suitability, the multi-epitope vaccine constructs were finalized and subjected to modelling and structural validation for further analyses.

#### 2.7.1. Modeling and Structural Validation

The 3-D structure of the finalized peptide vaccine candidates was modeled as per our previous studies combining homology and ab initio approach [45,46]. Swiss-Model (https://swissmodel.expasy.org/ (accessed on 27 September 2022)), Robetta (https://robetta.bakerlab.org/ (accessed on 27 September 2022)) and Modeller were used for this purpose [47,48]. By determining the stereochemical characteristics of the residues falling in the permitted regions (>95%) of the Ramachandran plot having the least unfavorable angles with the highest overall model score, the models were validated using the MolProbity tool (http://molprobity.biochem.duke.edu/index.php (accessed on 30 September 2022)) and Procheck (https://saves.mbi.ucla.edu/ (accessed on 30 September 2022)) [49,50,51]. Using the backbone N-H S^2^ order parameter values of the vaccine constructs, the DynaMine online interface (https://bio2byte.be/dynamine/ (accessed on 1 October 2022)) calculated the relative residue-level backbone dynamics. Based on constrained atomic bond vector orientations, S^2^ values represented experimental NMR chemical shifts. S^2^ values of >0.8 depict rigid conformations, while lower values indicate flexibility [52]. SOPMA web tool (https://npsa-prabi.ibcp.fr/cgi-bin/npsa_automat.pl?page=/NPSA/npsa_sopma.html (accessed on 1 October 2022)) was used to determine the peptides’ secondary structural patterns [53]. The most suitable vaccine construct was finalized based on model quality profiles.

#### 2.7.2. Target Retrieval and Optimization

The RCSB-Protein Data Bank was used to retrieve the target proteins 1H15 (HLA-DR B501:01), 2FSE (HLA-DR B101:01), 1A6A (HLA-DR B103:01), 2SEB (HLADRB1 04:01), 3C5J (HLA-DRB302:02), 2Q6W (HLA-DR B3 01:01) and 2Z65 (Human-Toll-Like receptor-4). Swiss-PDB viewer (SPDBV) was used to screen these six unique HLA alleles and toll-like receptor (TLR) alleles for missing residues and undesired hetero-atoms [54]. The protein structures were refined using the GalaxyRefine server (https://galaxy.seoklab.org/ (accessed on 1 October 2022)) by reducing clash scores, poor rotamers, percentage Ramachandran outliers and minimizing side-chain rotamers [55]. Finally, 2000 steps of the steepest descent and conjugate gradient algorithms using SPDBV with the GROMOS96 43B1 force-field in vacuo were used to minimize the energy of the structures. Thus, the target protein architectures were improved as per earlier studies [18,56,57].

#### 2.7.3. Target-Vaccine Constructs Docking

The vaccine construct was finally tested for interactions with six different HLA alleles and TLR-4 using the PatchDock web interface (https://bioinfo3d.cs.tau.ac.il/PatchDock/ (accessed on 3 October 2022)) [58]. PatchDock’s algorithm comprises segmented detection of target geometric patches, pose-clustering matching and complementarity scoring. Furthermore, the FireDock (https://bioinfo3d.cs.tau.ac.il/FireDock/ (accessed on 3 October 2022)) extension (rapid interaction refinement in molecular docking) was adopted, which provided the top 10 best solutions for final refinements based on global binding energy, atomic contact energies and intermolecular bonding contributors. The PatchDock docked complexes were therefore improved and re-scored [59]. The global binding energy and strong intermolecular interactions of the ‘target-vaccine complex’ models were further validated through Molecular Dynamics (MD) assessment. The docked complexes’ structures and intermolecular interactions were visualized with Chimera and LigPlot+, respectively [60,61].

### 2.8. Coarse-Grained Dynamics Simulation of the ‘Target-Vaccine Complex’ Model

The CABSflex interface (http://biocomp.chem.uw.edu.pl/CABSflex2 (accessed on 4 October 2022)) was employed to calculate the residue-level fluctuations of the target-vaccine complexes. The Root Mean Square Fluctuation (RMSF) trajectories, which were produced based on default restraint parameters, were used to determine the residue-level propensity, signifying the most advantageous conformation. Using an all-atom force-field-based MD simulation, the consensus protein variations in an aqueous environment were determined (10 nanosecond timescale). The default mode was selected, with minimum and maximum conformational distances of 3.8 and 8.0, respectively, and gap = 3 (minimum distance between the previous and next amino acid in the chain to be restrained). The relative atomic fluctuations were plotted to validate the most stable docked complex.

## 3. Results

### 3.1. Core vs. Pan-Genome Analysis and Vaccine Target Identification

**Figure 1:**
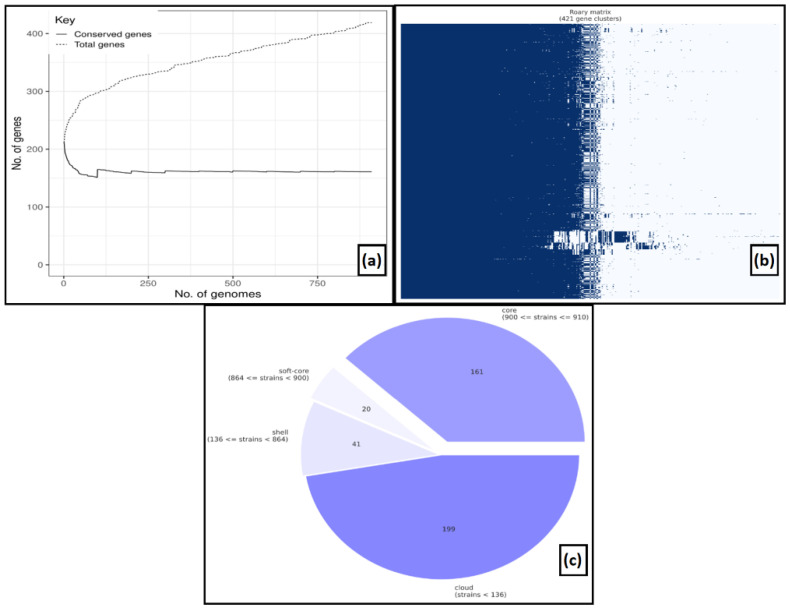
**Pan-genome analysis** (**a**) The number of conserved genes vs. total genes, (**b**) gene clusters, (**c**) pie-chart representing a number of core genes, soft-genes, shell, and cloud genes.

We generated a pan-genome based on the 910 annotation files of MPXV genomes to attain a higher sensitivity in detecting gene clusters. Comparing the core and pan-genomes, it was revealed that there were 161 core genes presented in all the MPXV genomes among 421 gene clusters with a 90% cut-off. Along with the core genes, the pan-genome comprised 20 soft-core genes in 864 isolates, 41 shell genes in 136 isolates and 199 genes in less than 136 isolates (Figure 1). 

With a 100% cut-off, the number of core genes was lowered to 102. These core genes with 100% cut-off were used for vaccine target mining. Then BLASTp was performed for 102 core genes to identify the non-homologous proteins against the human host proteome. The BLASTp screening suggested that there were 78 non-homologous proteins in the human proteome. Furthermore, these 78 non-homologous proteins were checked for their subcellular localization, and it was found that only 14 proteins were OMPs as predicted by Virus-mPLoc. The antigenicity analysis for shortlisted OMPs revealed that 10 proteins were found to be antigenic through VaxiJen v2.0 and ANTIGENpro. Upon analyzing the solubility of the filtered OMPs, it was observed that five OMPs were soluble in nature. Among five soluble OMPs, only three proteins, L5L, A28, and L5 (UniProt ID: Q77HN1, Q8V4U9 and Q8V4Z8), were antigenic and treated as vaccine candidates for further analysis. The results obtained from VaxiJen v2.0, ANTIGENpro and SolPro are given in Table 1.

### 3.2. Identification of Immunodominant Epitopes from Antigenic Proteins

#### 3.2.1. MHC Class-I T-Cell Epitope Mining

The MHC class-I T-cell epitopes were identified by subjecting the three antigenic protein sequences to ProPred1. It resulted in the identification of 61, 70 and 557 epitopes in L5L, A28 and L5 proteins, respectively. In addition to ProPred1, we submitted antigenic protein sequences in IEDB. From IEDB, a total of 26,331 epitopes were found. After considering of cut-off IC50 values < 50 nM, 18, 27 and 39 unique epitopic sequences were identified in L5L, A28 and L5 proteins, respectively. In order to increase the efficacy of our vaccine constructs, we chose the epitopes commonly (n = 42) presented in proteins from both tools and tested for their immunogenicity. The IEDB immunogenicity analysis revealed, out of 42, only 23 epitopes as most immunogenic epitopes. Additionally, these 23 epitopes were evaluated for their toxicity and antigenicity, and their scores are provided in Table 2. It was noted that all 23 epitopes were non-toxic, and only 12 epitopes were found to be antigenic as well as immunogenic. Thus, we considered these 12 MHC class-I T-cell epitopes for our vaccine construction by excluding the remaining epitopes from our analysis.

#### 3.2.2. MHC Class-II T-Cell Epitope Mining

The MHC class-II T-cell epitopes were identified by the IEDB server, which generated a total of 23,779 epitopes in three shortlisted proteins. With percentile rank < 0.2 and IC50 < 50 nM, the L5L, A28, and L5 proteins contained unique 4, 33 and 67 MHC class-II T-cell epitopes, respectively. These were considered as good binders to MHC class-II molecules. Subsequently, we checked immunogenicity for these good binders by CD4+ T-cell immunogenicity prediction tool and observed that only five epitopic sequences presented in the L5 protein were immunogenic with percentile rank cut-off of <66. The immunogenic MHC class-II T-cell epitopic sequences in the other two proteins, L5L and A28, were absent. Moreover, the five epitopic sequences in the L5 protein were non-toxic and antigenic, as predicted by ToxinPred and VaxiJen v2.0 tool. Hence, they were considered for our vaccine construction. The immunogenicity, antigenicity, and toxicity of the finalized MHC class-II T-cell epitopes are given in Table 3. 

#### 3.2.3. MHC Restricted Alleles Cluster Analysis

**Figure 2:**
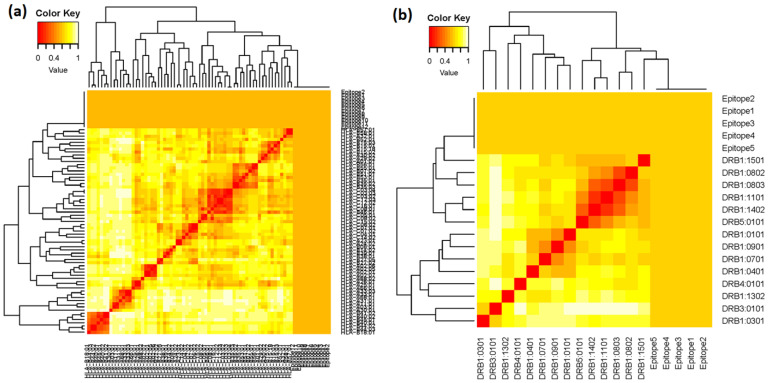
**MHC clustering based on binding specificity:** (**a**) heat map of 12 MHC class-I T-cell epitopes, and (**b**) heat map of 5 MHC class-II T-cell epitopes.

MHC class-I and MHC class-II alleles were further investigated based on their IC50 value. All the finalized epitopic sequences (12 MHC class-I T-cell epitopes and 5 MHC class-II T-cell epitopes) were independently evaluated for their MHC interaction analysis using MHCcluster v2.0. The epitopic sequences were clustered by interaction with HLA, and the interacted alleles were shown as a heat map (Figure 2). In the heat map, the stronger and weaker interactions are represented in red and yellow respectively, along with proper annotation.

#### 3.2.4. Selection of B-Cell Epitope for Vaccine Construction

**Figure 3:**
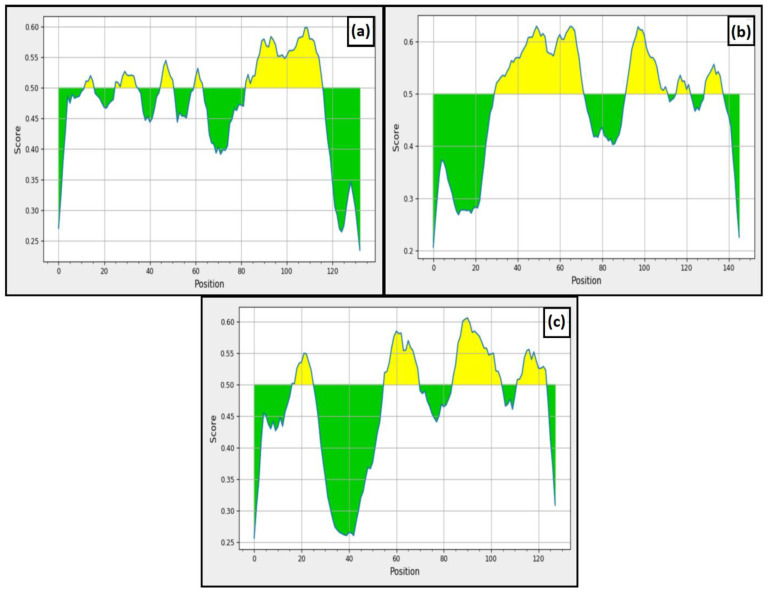
**BepiPred linear epitope prediction** (**a**) L5L, (**b**) A28, and (**c**) L5.

Along with cellular immunity, humoral immunity is also responsible for eliminating the pathogen from the host. Our study used BepiPred v2.0 and ABCPred to predict the B-cell epitopes. From ABCPred, 10, 14 and 9 B-cell epitopic sequences were found in L5L, A28 and L5 proteins, respectively. In addition, the resultant epitopes were further scrutinized based on the BepiPred v2.0 (Figure 3). In the figure, the yellow area represents the region with good scores that have a high probability of acting as B-cell epitopes. From BepiPred v2.0, five epitopes were found in L5L, whereas four epitopes were present in A28 and L5 protein. Finally, we compared the results achieved from both the tools to finalize the similar epitopes predicted through these tools. The results revealed that only 10 B-cell epitopes were similar among all these predicted epitopes and further used for vaccine construction. 

### 3.3. Vaccine Construction

The overlapping and distinctive epitopes were chosen for vaccine construction from the shortlisted MHC class-I, MHC class-II T-cell epitopes, and linear B-cell epitopes (Table 4).

**Figure 4:**
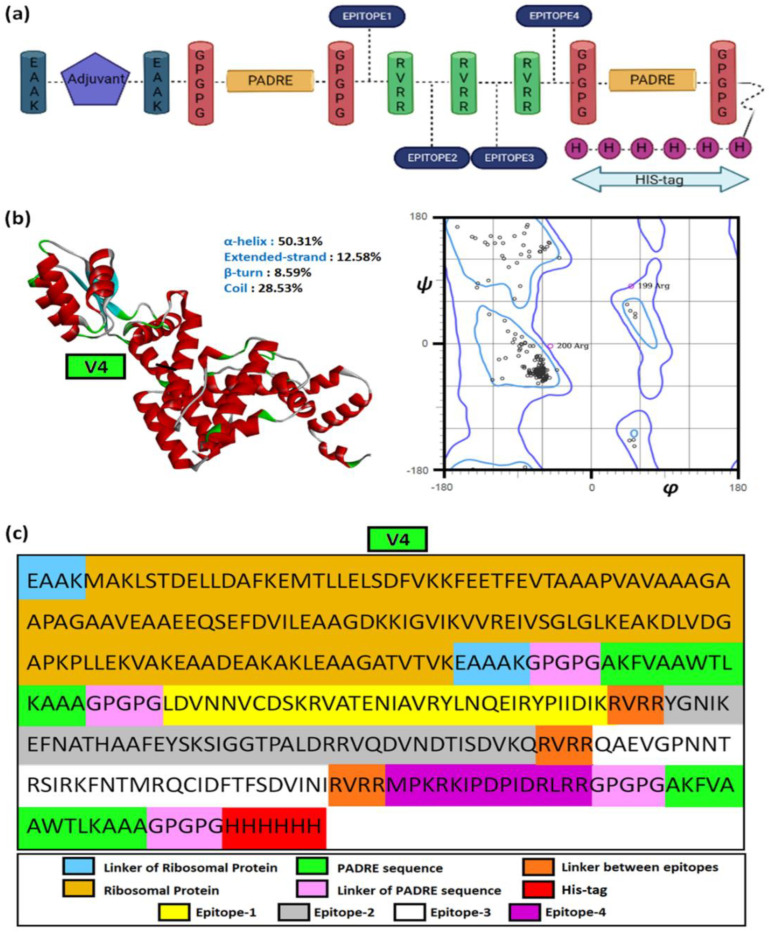
**The vaccine construct**. (**a**) The general structural outline of the vaccine constructs, (**b**) the structure of vaccine construct 4 (V4) with secondary structural components and corresponding Ramachandran plot, and (**c**) the detailed structural constituents of V4.

Hence, the overlapping epitopes would have the ability to induce both innate and humoral immunity. This study used β-defensin, heparin-binding haemagglutinin (HBHA), HBHA conserved and L7/L12 ribosomal proteins as the adjuvants. The epitopes were placed in various combinations after adjuvant. The linkers EAAK, GPGPG, and RVRR were used to separate the adjuvants, PADRE sequence, and epitopic sequences, respectively. The EAAK linkers were added in the N and C terminals of the adjuvants. These linkers are immunogenicity boosters. The PADRE sequence was added in order to increase the efficacy of the vaccine by stimulating CD4+ cells. Finally, His Tag was added to the C-terminal to form the final vaccine construct. The classical form of vaccine constructs is shown in Figure 4a. A total of 16 vaccine constructs (V1-V16) were prepared with different combinations of adjuvants and linkers and these are given in Table 5. 

### 3.4. Allergicity, Antigenicity, Solubility and Physio-Chemical Parameters Evaluation of Vaccine Constructs

The designed vaccine constructs were then assessed for their properties to be considered as an effective vaccine. The allergenicity, antigenicity and physio-chemical properties of 16 vaccine constructs are given in Table 6. 

Foremost, the vaccine constructs were tested for allergenicity, and it was found that all our vaccine constructs were non-allergen. Upon checking for their antigenicity, it was observed that V6, V10 and V14 were non-antigenic in nature. Thus, we excluded these vaccine constructs from our study. The physio-chemical properties were calculated for the remaining vaccine constructs by the ProtParam tool. All vaccine constructs showed high solubility, and their molecular weight ranged from 26–39 kDa, with pI score of 9.11–10.40. The GRAVY value ranging between −0.2 and −0.6 can elicit a good immunogenic response. While comparing the stability among vaccine constructs, it was noted that only V4, V8, V12, and V16 were more stable compared to other vaccine constructs. Thus, we finalized these four predicted vaccine constructs for our study and further evaluated them through structural parameters. 

### 3.5. Modelling, Docking and Structural Dynamics to Evaluate the Vaccine Constructs

#### 3.5.1. Multi-Epitope Vaccine Construct Models

A combined approach comprising homology and ab initio approaches was used to model the vaccine constructs, followed by structural validations. The final model or the most suitable vaccine construct, i.e., V4, was chosen based on the highest Ramachandran favored region (96.06%) and overall quality factor (99.05). V4 possessed marginally lower backbone rigidity than V8, V12 and V16. The relative structural properties of the models are given in Table 7. 

The average S^2^-scores depicting backbone flexibility also showed V4 having marginally lesser rigidity (0.783) than the other vaccine constructs (0.785) in the multi-epitopic domain. The residue-wise S^2^-scores are given in Supplementary File-S1. Figure 4b,c depicts the structural model of V4 and its components. 

#### 3.5.2. Docking Studies to Reveal the Binding Affinity of the Suitable Construct

The docked complexes of V4 depicted its selective affinity towards different HLA-alleles and TLR-4. According to the lowest global binding energy, V4 possessed the highest affinity towards two HLA-alleles, 1H15 (−23.62 kcal/mol) and 2FSE (−17.38 kcal/mol) besides TLR-4 or 2Z65 (−1.98 kcal/mol). The same was contributed by attractive van der Waal forces, atomic contact energies and hydrogen bonds (Table 8). 

**Figure 5:**
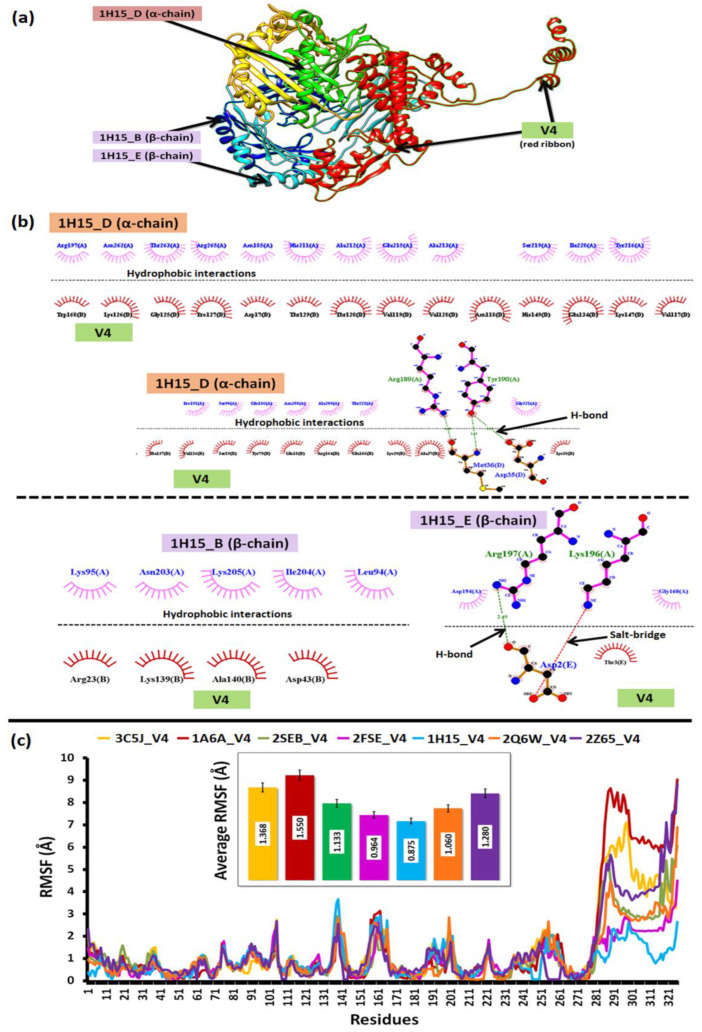
**The docking and dynamics profile of V4** shows (**a**) V4 complex with 1H14, (**b**) inter-molecular interactions between the V4-1H14 complex, and (**c**) the relative structural dynamics profile of the V4 complex with all the immune targets.

The strong hydrophobic interaction between 26 amino acid residues of the V4 with 29 residues of the α and β-chains of the target 1H15, besides four hydrogen bonds (Arg189, Tyr190, Arg197) and one salt-bridge (Lys196), contributed to its superior binding profiles, as given in Figure 5a,b.

#### 3.5.3. Structural Dynamics to Reveal the Stability of the Complexes

The relative dynamics of V4 interacting with the HLA-alleles and TLR-4 validated the docking profiles. It was observed that RMSF derived from the coarse-grained simulations suggested a relative low difference in average fluctuations (<1 Å) among the docked complexes. However, the average fluctuations of V4 while interacting with 1H15 (0.875) and 2FSE (0.964) were the lowest as compared to the rest, which further validated their low binding energy (from docking) and stable conformation. The relative average RMSF values are illustrated in Figure 5c.

## 4. Discussion

MPXV is a dsDNA orthopoxvirus causing emerging and re-emerging outbreaks in various parts of the world, with increasing mortality rates in children and immunocompromised people [2,3]. The dearth of specific vaccines or drug candidates against MPXV has imposed alarming threats due to its evolving pathogenic traits [4]. Vaccines are the most effective and cost-efficient solutions to eliminate viral outbreaks. An effective vaccine should have the ability to induce humoral and cell-mediated immunity against the target viral infected cells [62]. Hence, a genome-based reverse vaccinology approach was adopted to design extensively validated multi-epitopic vaccine candidates that can efficiently provide immunity against MPXV infections.

Since immunoinformatics approaches are used as popular first-line vaccine design techniques, there have been a few notable studies on the same approach adding to the quest to design suitable multi-epitopic MPXV vaccines [8,63,64,65]. Although each study stands out in their meticulous screening of targets and unique vaccine constructs, the context of involving pan-genome has not been explored earlier. Pan-genome-based screening establishes the global uniformity of vaccine targets in MPXV and eliminates the limitations (biases) of data acquired from secondary/curated sources. More than 2000 whole genomes identified worldwide were considered for the study, to funnel down to a set of 910 available annotated genomes to extract vaccine targets. From 910 annotated whole genomes of MPXV, potential target proteins were screened based on heterology to human proteins, solubility, outer-membrane location, and antigenicity. The conservation properties ensured minimal cross-reactivity while eliciting immune responses. MPXV OMPs L5L, A28 and L5 were finalized as potential vaccine targets. Furthermore, the structural analyses and docking studies encompassed all potential human targets (HLA and TLR) to comprehensively understand the specific immunological response against MPXV. 

Immunogenicity can be induced by coupling B-cell and T-cell epitopes. The humoral immune response elicited by B-lymphocytes comprises pathogen-specific antibodies that can effectively combat viral loads by neutralizing the antigenic determinants. On the other hand, cytotoxic T-cells (CTLs) activated by MHC class-I epitopes can engender long-lasting protection against virus-infected cells [66,67,68]. In the present study, stringent screening through combined immunogenic scores, non-toxic nature and adherence to MHC qualities were taken into account while predicting the epitopes. Furthermore, the vaccine components were manually adjusted to design a suitable vaccine construct of high immunogenicity and structural stability.

Cellular immunity plays an influential role against viral infections, activating CD4+ T-cells, CD8+ T-cells and B-cells [32]. Outer-membrane proteins in MPXV have been reported earlier to be responsible for host attachment, invasion, and pathogenesis besides being unique to the strains. Hence, the OMPs of MPXV was finalized thereafter to extract MHC class-I T-cell epitopes, MHC class-II T-cell epitopes and B-cell epitopes, which can trigger effective immune responses after binding. The same was validated from various server-based predictions. The final vaccine construct was designed by aligning multiple epitopes and connecting them with suitable linkers, adjuvants, PADRE sequences and His Tags to enhance the efficacy. The use of adjuvants in the vaccine construction makes the vaccine able to promote and stimulate the stronger protective immune response in people receiving the vaccine. We used HBHA, HBHA conserved, β-defensin and L7/L12 ribosomal proteins as adjuvants in the present study. Among these adjuvants, β-defensin plays a major role in innate immunity and is an antimicrobial peptide. In order to induce the immune response by β-defensin, it functions with the help of dendritic cells, monocytes and T-cells. The innate immune system safeguards the host from microbial diseases, such as bacteria, viruses, parasites and fungi, as the first-line of defense [69]. The other adjuvant was L7/L12 ribosomal protein that actively participates in the start, elongation, and termination of translation process and is essential for elongation factor-GTPase activity. HBHA protein adjuvant is an immunodominant antigen that stimulates T-cells and aids in the production of interferon-gamma production [70,71]. In addition to the adjuvants, the use of linkers can also boost the immunogenicity while the PADRE sequence assists in the activation of CD4+ cells. The alpha helix forming linkers with the sequence of (EAAAK) was added at the C and N-terminal of adjuvants. As proposed by George and Heringa, many natural linkers exhibited α-helical structures [72]. The α-helical structures are more rigid and stable, along with the segment of hydrogen bonds and a closely packed backbone. Therefore, the stiff α-helical linkers have been considered as rigid spacers between the adjuvants [73].

The set of 16 vaccine constructs were prioritized out of various combinations based on immunological complementarity and chemical stability. The finalized vaccine constructs (V4, V8, V12 and V16) were validated with docking and dynamics analyses. All the finalized vaccine constructs have L7/L12 ribosomal proteins as adjuvants, which are more stable than other vaccine constructs with β-defensin, HBHA and HBHA conserved proteins. Firstly, the chosen constructs were modelled using a combined extended modelling approach to resolve ambiguities concerning low-compatibility (much less sequence identity/coverage) modelling templates. The most suitable construct, i.e., V4, represented high-quality structural profiles and backbone dynamics to be considered for further analyses. The relatively low backbone rigidity (S^2^-score < 0.8) of V4 over the others was favorable for it to establish transient interactions with its HLA-targets [18,45]. The high-binding affinity (>−15 kcal/mol) towards HLA-molecules was facilitated by intra-molecular salt-bridges, hydrogen bonds and hydrophobic contacts. The threshold of high/higher binding energy values was interpreted from previous literature on reverse vaccinology [12]. The relative dynamics of V4 with HLA class-II Histocompatibility Antigen (PDB IDs: 1H15, 2FSE) further supported the docking studies by portraying structural stability having low atomic level fluctuations (<1 Å). The fluctuations (and greater flexibility) in the epitope-containing domain of the constructs can be due to the higher number of weak interactions formed with this domain, as seen in Figure 5b,c. 

Although the computational screening justified our objectives, the major limitation of the study comprises multiple predictions, which will have a meaningful translational benefit post experimental validation. Hence, the present study and similar computational vaccine design studies are likely to enhance the vaccine regimen against MPXV (and similar pathogens) to encourage extensive in vitro/in vivo research and development.

## 5. Conclusions

Emerging and re-emerging MPVX infections have threatened the healthcare sector due to evolving genomic traits and a lack of targeted therapy. The present study endeavored to screen potential vaccine targets through extensive pangenome analyses followed by epitope identification and designing suitable multi-epitopic vaccine constructs. Immunologically compatible and safe vaccine constructs established high affinity and stability towards interacting with human HLA-proteins and TLR-4. A potent vaccine construct was finally designed to encourage future experimental evaluations.

## Figures and Tables

**Table 1:** Antigenicity and solubility of 14 OMPs.

Protein Name	UniProt ID	AntigenPro	VaxiJen		SolPro	
		Predictefud Probability of Antigenicity	Antigenicity	Overall Protective Probable Antigen Prediction	Solubility upon Overexpression	Probability
Un-characterized protein	Q3I962	0.197732	Probable Non-Antigen	0.3865	Soluble	0.953785
L5L	Q77HN1	0.629557	Probable Antigen	0.6787	Soluble	0.605143
B20R	Q8V4Q8	0.892397	Probable Antigen	0.6077	Insoluble	0.905405
A35R	Q8V4U4	0.972597	Probable Antigen	0.5392	Insoluble	0.734623
M1R	Q8V502	0.940711	Probable Antigen	0.6136	Insoluble	0.689408
Protein L5	Q8V4Z8	0.352401	Probable Antigen	0.4607	Soluble	0.531955
C15L	Q8V539	0.52584	Probable Antigen	0.4806	Insoluble	0.850441
A5L	Q8V4 × 1	0.942329	Probable Antigen	0.4029	Insoluble	0.867695
Protein G3	Q8V509	0.523446	Probable Non-Antigen	0.356	Insoluble	0.828577
A28	Q8V4U9	0.753088	Probable Antigen	0.5530	Soluble	0.656282
C23R	Q8V531	0.637633	Probable Non-Antigen	0.3572	Soluble	0.792761
C11L	Q8V543	0.818476	Probable Antigen	0.5360	Insoluble	0.68359
A18L	Q8V4W0	0.139456	Probable Non-Antigen	0.2683	Insoluble	0.690301
O2L	Q8V554	0.830181	Probable Antigen	0.7249	Insoluble	0.817524

**Table 2:** **The immunogenicity, antigenicity, and toxicity of 23 MHC class-I T-cell epitopes**. Highlighted (bold) are finalized MHC class-I T-cell epitopes.

Protein Name	UniProt ID	Predicted Epitopes	Immunogenicity	Antigenicity	Toxicity
L5L	Q77HN1	RVATENIAV	0.27835	−0.0904	Non-Toxin
		** ALAILILAF **	** 0.2746 **	** 0.6926 **	** Non-Toxin **
		** GLLALAILI **	** 0.16764 **	** 0.7314 **	** Non-Toxin **
		** ATENIAVRY **	** 0.2575 **	** 0.4092 **	** Non-Toxin **
A28	Q8V4U9	** NSLSIFFIV **	** 0.24629 **	** 0.5713 **	** Non-Toxin **
		** FIVVATAAV **	** 0.18546 **	** 0.6997 **	** Non-Toxin **
		** KEFNATHAA **	** 0.15629 **	** 1.079 **	** Non-Toxin **
		** NATHAAFEY **	** 0.27738 **	** 1.2587 **	** Non-Toxin **
		** KQKWRCVVY **	** 0.2115 **	** 1.7426 **	** Non-Toxin **
		** SIFGFQAEV **	** 0.16858 **	** 0.4227 **	** Non-Toxin **
		** MRQCIDFTF **	** 0.18011 **	** 1.7234 **	** Non-Toxin **
L5	Q8V4Z8	FEVFVVFIL	0.38682	0.1235	Non-Toxin
		IVLFEVFVV	0.37348	0.0325	Non-Toxin
		** NPVFIEPTF **	** 0.36837 **	** 1.369 **	** Non-Toxin **
		VFVVFILIY	0.36262	0.2344	Non-Toxin
		FVVFILIYV	0.36052	0.347	Non-Toxin
		VLFEVFVVF	0.34811	0.0364	Non-Toxin
		EVFVVFILI	0.33578	0.1919	Non-Toxin
		FILIYVFFR	0.33278	0.21	Non-Toxin
		RLIVLFEVF	0.29276	−0.2760	Non-Toxin
		VFILIYVFF	0.2614	0.0066	Non-Toxin
		** NVYFNPVFI **	** 0.2031 **	** 1.4789 **	** Non-Toxin **
		LMIYGLPWI	0.1821	0.388	Non-Toxin

**Table 3:** The immunogenicity, antigenicity, and toxicity of finalized MHC class-II T-cell epitopes.

Protein Name	UniProt ID	Predicted Epitopes	Immunogenicity	Antigenicity	Toxicity
L5	Q8V4Z8	FFRSELNMFFMPKRK	56.1564	0.7973	Non-Toxin
		FRSELNMFFMPKRKI	58.0094	0.5608	Non-Toxin
		SELNMFFMPKRKIPD	51.2572	0.5064	Non-Toxin
		ELNMFFMPKRKIPDP	56.1681	0.6630	Non-Toxin
		LNMFFMPKRKIPDPI	53.3717	0.6685	Non-Toxin

**Table 4:** Combined results of MHC class-I T-cell, MHC class-II T-cell and B cell epitope prediction in three filtered proteins.

Protein Name	MHC Class-I T-Cell		MHC Class-II T-Cell		B-Cell	
	Predicted Epitope	Position	Predicted Epitope	Position	Predicted Epitope	Position
L5L	ALAILILAF	124–132	-	-	NKDD	13–16
	GLLALAILI	121–129	-	-	KSIVRIGIDTR	26–36
	ATENIAVRY	95–103	-	-	ISRC	61–64
	-	-	-	-	LDVNNVCDSKRVATENIAVRYLNQEIRYPIIDIK	83–116
A28	NSLSIFFIV	2–10	-	-	QAEVGPNNTRSIRKFNTMRQ	92–111
	FIVVATAAV	8–16	-	-	FSDVINI	117–123
	KEFNATHAA	34–42	-	-	YGNIKEFNATHAAFEYSKSIGGTPALDRRVQDVNDTISDVKQ	30–71
	NATHAAFEY	37–45	-	-	-	-
	KQKWRCVVY	70–78	-	-	-	-
	SIFGFQAEV	87–95	-	-	-	-
	MRQCIDFTF	109–117	-	-	-	-
L5	NVYFNPVFI	6–14	FFRSELNMFFMPKRK	46–60	KHSLLSVY	19–26
	NPVFIEPTF	10–18	FRSELNMFFMPKRKI	47–61	MPKRKIPDPIDRLRR	56–70
	-	-	SELNMFFMPKRKIPD	49–63	LPWITTQTSALSINSKPIVYK	85–105
	-	-	ELNMFFMPKRKIPDP	50–64	-	-
	-	-	LNMFFMPKRKIPDPI	51–65	-	-

**Table 5:** Different combinations of vaccine constructs.

Vaccine Construct	Vaccine Composition	Sequence
V1	HBHA, E1, E2, E3, E4, linkers, PADRE, His Tag	EAAKMAENPNIDDLPAPLLAALGAADLALATVNDLIANLRERA EETRAETRTRVEERRARLTKFQEDLPEQFIELRDKFTTEELRKAAE GYLEAATNRYNELVERGEAALQRLRSQTAFEDASARAEGYVDQA VELTQEALGTVASQTRAVGERAAKLVGIELEAAKGPGPGAKFVA AWTLKAAAGPGPGLDVNNVCDSKRVATENIAVRYLNQEIRYPII DIKRVRRYGNIKEFNATHAAFEYSKSIGGTPALDRRVQDVNDTIS DVKQRVRRQAEVGPNNTRSIRKFNTMRQCIDFTFSDVINIRVRRM PKRKIPDPIDRLRRGPGPGAKFVAAWTLKAAAGPGPGHHHHHH
V2	HBHA conserved, E1, E2, E3, E4, linkers, PADRE, His Tag	EAAAKMAENSNIDDIKAPLLAALGAADLALATVNELITNLRERA EETRRSRVEESRARLTKLQEDLPEQLTELREKFTAEELRKAAEGYL EAATSELVERGEAALERLRSQQSFEEVSARAEGYVDQAVELTQEA LGTVASQVEGRAAKLVGIELEAAAKGPGPGAKFVAAWTLKAAA GPGPGLDVNNVCDSKRVATENIAVRYLNQEIRYPIIDIKRVRRYG NIKEFNATHAAFEYSKSIGGTPALDRRVQDVNDTISDVKQRVRRQ AEVGPNNTRSIRKFNTMRQCIDFTFSDVINIRVRRMPKRKIPDPID RLRRGPGPGAKFVAAWTLKAAAGPGPGHHHHHH
V3	β-Defensin, E1, E2, E3, E4, linkers, PADRE, His Tag	EAAKGIINTLQKYYCRVRGGRCAVLSCLPKEEQIGKCSTRGRKCC RRKKEAAKGPGPGAKFVAAWTLKAAAGPGPGLDVNNVCDSKR VATENIAVRYLNQEIRYPIIDIKRVRRYGNIKEFNATHAAFEYSKSI GGTPALDRRVQDVNDTISDVKQRVRRQAEVGPNNTRSIRKFNTM RQCIDFTFSDVINIRVRRMPKRKIPDPIDRLRRGPGPGAKFVAAWT LKAAAGPGPGHHHHHH
V4	L7/L12 Ribosomal protein, E1, E2, E3, E4, linkers, PADRE, His Tag	EAAKMAKLSTDELLDAFKEMTLLELSDFVKKFEETFEVTAAAPVA VAAAGAAPAGAAVEAAEEQSEFDVILEAAGDKKIGVIKVVREIVS GLGLKEAKDLVDGAPKPLLEKVAKEAADEAKAKLEAAGATVTV KEAAAKGPGPGAKFVAAWTLKAAAGPGPGLDVNNVCDSKRVA TENIAVRYLNQEIRYPIIDIKRVRRYGNIKEFNATHAAFEYSKSIGG TPALDRRVQDVNDTISDVKQRVRRQAEVGPNNTRSIRKFNTMRQ CIDFTFSDVINIRVRRMPKRKIPDPIDRLRRGPGPGAKFVAAWTLK AAAGPGPGHHHHHH
V5	HBHA, E2, E1, E3, E4, linkers, PADRE, His Tag	EAAKMAENPNIDDLPAPLLAALGAADLALATVNDLIANLRERA EETRAETRTRVEERRARLTKFQEDLPEQFIELRDKFTTEELRKAAE GYLEAATNRYNELVERGEAALQRLRSQTAFEDASARAEGYVDQ AVELTQEALGTVASQTRAVGERAAKLVGIELEAAKGPGPGAKFV AAWTLKAAAGPGPGYGNIKEFNATHAAFEYSKSIGGTPALDRRV QDVNDTISDVKRVRRLDVNNVCDSKRVATENIAVRYLNQEIRYPI IDIKRVRRQAEVGPNNTRSIRKFNTMRQCIDFTFSDVINIRVRRMPK RKIPDPIDRLRRGPGPGAKFVAAWTLKAAAGPGPGHHHHHH
V6	HBHA conserved, E2, E1, E3, E4, linkers, PADRE, His Tag	EAAAKMAENSNIDDIKAPLLAALGAADLALATVNELITNLRERA EETRRSRVEESRARLTKLQEDLPEQLTELREKFTAEELRKAAEGYL EAATSELVERGEAALERLRSQQSFEEVSARAEGYVDQAVELTQEA LGTVASQVEGRAAKLVGIELEAAAKGPGPGAKFVAAWTLKAAA GPGPGYGNIKEFNATHAAFEYSKSIGGTPALDRRVQDVNDTISDV KRVRRLDVNNVCDSKRVATENIAVRYLNQEIRYPIIDIKRVRRQA EVGPNNTRSIRKFNTMRQCIDFTFSDVINIRVRRMPKRKIPDPIDRLR RGPGPGAKFVAAWTLKAAAGPGPGHHHHHH
V7	β-Defensin, E2, E1, E3, E4, linkers, PADRE, His Tag	EAAKGIINTLQKYYCRVRGGRCAVLSCLPKEEQIGKCSTRGRKCC RRKKEAAKGPGPGAKFVAAWTLKAAAGPGPGYGNIKEFNATH AAFEYSKSIGGTPALDRRVQDVNDTISDVKRVRRLDVNNVCDSK RVATENIAVRYLNQEIRYPIIDIKRVRRQAEVGPNNTRSIRKFNTM RQCIDFTFSDVINIRVRRMPKRKIPDPIDRLRRGPGPGAKFVAAWT LKAAAGPGPGHHHHHH
V8	L7/L12 Ribosomal protein, E2, E1, E3, E4, linkers, PADRE, His Tag	EAAKMAKLSTDELLDAFKEMTLLELSDFVKKFEETFEVTAAAPVA VAAAGAAPAGAAVEAAEEQSEFDVILEAAGDKKIGVIKVVREIVS GLGLKEAKDLVDGAPKPLLEKVAKEAADEAKAKLEAAGATVTV KEAAAKGPGPGAKFVAAWTLKAAAGPGPGYGNIKEFNATHAA FEYSKSIGGTPALDRRVQDVNDTISDVKRVRRLDVNNVCDSKRVA TENIAVRYLNQEIRYPIIDIKRVRRQAEVGPNNTRSIRKFNTMRQCI DFTFSDVINIRVRRMPKRKIPDPIDRLRRGPGPGAKFVAAWTLKA AAGPGPGHHHHHH
V9	HBHA, E3, E2, E1, E4, linkers, PADRE, His Tag	EAAKMAENPNIDDLPAPLLAALGAADLALATVNDLIANLRERA EETRAETRTRVEERRARLTKFQEDLPEQFIELRDKFTTEELRKAAE GYLEAATNRYNELVERGEAALQRLRSQTAFEDASARAEGYVDQ AVELTQEALGTVASQTRAVGERAAKLVGIELEAAKGPGPGAKFV AAWTLKAAAGPGPGQAEVGPNNTRSIRKFNTMRQCIDFTFSDVI NIRVRRYGNIKEFNATHAAFEYSKSIGGTPALDRRVQDVNDTISD VKRVRRLDVNNVCDSKRVATENIAVRYLNQEIRYPIIDIKRVRRM PKRKIPDPIDRLRRGPGPGAKFVAAWTLKAAAGPGPGHHHHHH
V10	HBHA conserved, E3, E2, E1, E4, linkers, PADRE, His Tag	EAAAKMAENSNIDDIKAPLLAALGAADLALATVNELITNLRERA EETRRSRVEESRARLTKLQEDLPEQLTELREKFTAEELRKAAEGYL EAATSELVERGEAALERLRSQQSFEEVSARAEGYVDQAVELTQEA LGTVASQVEGRAAKLVGIELEAAAKGPGPGAKFVAAWTLKAAA GPGPGQAEVGPNNTRSIRKFNTMRQCIDFTFSDVINIRVRRYGNIK EFNATHAAFEYSKSIGGTPALDRRVQDVNDTISDVKRVRRLDVN NVCDSKRVATENIAVRYLNQEIRYPIIDIKRVRRMPKRKIPDPIDRL RRGPGPGAKFVAAWTLKAAAGPGPGHHHHHH
V11	β-Defensin, E3, E2, E1, E4, linkers, PADRE, His Tag	EAAKGIINTLQKYYCRVRGGRCAVLSCLPKEEQIGKCSTRGRKCC RRKKEAAKGPGPGAKFVAAWTLKAAAGPGPGQAEVGPNNTRSI RKFNTMRQCIDFTFSDVINIRVRRYGNIKEFNATHAAFEYSKSIGG TPALDRRVQDVNDTISDVKRVRRLDVNNVCDSKRVATENIAVRY LNQEIRYPIIDIKRVRRMPKRKIPDPIDRLRRGPGPGAKFVAAWTL KAAAGPGPGHHHHHH
V12	L7/L12 Ribosomal protein, E3, E2, E1, E4, linkers, PADRE, His Tag	EAAKMAKLSTDELLDAFKEMTLLELSDFVKKFEETFEVTAAAPVA VAAAGAAPAGAAVEAAEEQSEFDVILEAAGDKKIGVIKVVREIVS GLGLKEAKDLVDGAPKPLLEKVAKEAADEAKAKLEAAGATVTV KEAAAKGPGPGAKFVAAWTLKAAAGPGPGQAEVGPNNTRSIRK FNTMRQCIDFTFSDVINIRVRRYGNIKEFNATHAAFEYSKSIGGTP ALDRRVQDVNDTISDVKRVRRLDVNNVCDSKRVATENIAVRYL NQEIRYPIIDIKRVRRMPKRKIPDPIDRLRRGPGPGAKFVAAWTLK AAAGPGPGHHHHHH
V13	HBHA, E4, E3, E2, E1, linkers, PADRE, His Tag	EAAKMAENPNIDDLPAPLLAALGAADLALATVNDLIANLRERA EETRAETRTRVEERRARLTKFQEDLPEQFIELRDKFTTEELRKAAE GYLEAATNRYNELVERGEAALQRLRSQTAFEDASARAEGYVDQ AVELTQEALGTVASQTRAVGERAAKLVGIELEAAKGPGPGAKFV AAWTLKAAAGPGPGMPKRKIPDPIDRLRRRVRRQAEVGPNNTRS IRKFNTMRQCIDFTFSDVINIRVRRYGNIKEFNATHAAFEYSKSIGG TPALDRRVQDVNDTISDVKRVRRLDVNNVCDSKRVATENIAVRY LNQEIRYPIIDIKGPGPGAKFVAAWTLKAAAGPGPGHHHHHH
V14	HBHA conserved, E4, E3, E2, E1, linkers, PADRE, His Tag	EAAAKMAENSNIDDIKAPLLAALGAADLALATVNELITNLRERA EETRRSRVEESRARLTKLQEDLPEQLTELREKFTAEELRKAAEGYL EAATSELVERGEAALERLRSQQSFEEVSARAEGYVDQAVELTQEA LGTVASQVEGRAAKLVGIELEAAAKGPGPGAKFVAAWTLKAAA GPGPGMPKRKIPDPIDRLRRRVRRQAEVGPNNTRSIRKFNTMRQC IDFTFSDVINIRVRRYGNIKEFNATHAAFEYSKSIGGTPALDRRVQ DVNDTISDVKRVRRLDVNNVCDSKRVATENIAVRYLNQEIRYPII DIKGPGPGAKFVAAWTLKAAAGPGPGHHHHHH
V15	Defensin, E4, E3, E2, E1, linkers, PADRE, His Tag	EAAKGIINTLQKYYCRVRGGRCAVLSCLPKEEQIGKCSTRGRKCC RRKKEAAKGPGPGAKFVAAWTLKAAAGPGPGMPKRKIPDPIDR LRRRVRRQAEVGPNNTRSIRKFNTMRQCIDFTFSDVINIRVRRYG NIKEFNATHAAFEYSKSIGGTPALDRRVQDVNDTISDVKRVRRLD VNNVCDSKRVATENIAVRYLNQEIRYPIIDIKGPGPGAKFVAAWT LKAAAGPGPGHHHHHH
V16	L7/L12 Ribosomal protein, E4, E3, E2, E1, linkers, PADRE, His Tag	EAAKMAKLSTDELLDAFKEMTLLELSDFVKKFEETFEVTAAAPVA VAAAGAAPAGAAVEAAEEQSEFDVILEAAGDKKIGVIKVVREIVS GLGLKEAKDLVDGAPKPLLEKVAKEAADEAKAKLEAAGATVTV KEAAAKGPGPGAKFVAAWTLKAAAGPGPGMPKRKIPDPIDRLR RRVRRQAEVGPNNTRSIRKFNTMRQCIDFTFSDVINIRVRRYGNIK EFNATHAAFEYSKSIGGTPALDRRVQDVNDTISDVKRVRRLDVN NVCDSKRVATENIAVRYLNQEIRYPIIDIKGPGPGAKFVAAWTLK AAAGPGPGHHHHHH

Green colored amino acids: linker of adjuvants; orange colored amino acids: adjuvant; blue colored amino acids: linker of PADRE sequence; red colored amino acids: PADRE sequence; yellow highlighted amino acids: epitope 1; cyan highlighted amino acids: epitope 2; magenta highlighted amino acids: epitope 3; green highlighted amino acids: epitope 4; purple colored amino acids: linker between epitopes; black colored amino acids: His Tag.

**Table 6:** Allergenicity, antigenicity, solubility and physio-chemical properties of predicted 16 vaccine constructs. (Negative values in red fonts were not considered further).

Vaccine	Probable Non-Allergen	Probable Antigen		Solubility Probability	Molecular Weight	Theoretical pI	Instability Index	Aliphatic Index	GRAVY
Server Used	AlgPred, AllergenFP 2.0	VaxiJen	AntigenPro	ProtParam	ProtParam	ProtParam	ProtParam	ProtParam	ProtParam
V1	+	+(0.4936)	+(0.668389)	+(0.871680)	39243.35	9.35	−(43.12)	80.90	−0.570
V2	+	+(0.4957)	+(0.602766)	+(0.920375)	38267.29	9.11	−(47.62)	83.92	−0.530
V3	+	+(0.5062)	+(0.732167)	+(0.979442)	26775.90	10.40	−(42.88)	71.58	−0.644
V4	+	+(0.4583)	+(0.719020)	+(0.857865)	35126.27	9.31	+(33.47)	85.12	−0.300
V5	+	+(0.5092)	+(0.521264)	+(0.857733)	39115.22	9.35	−(43.33)	81.13	−0.562
V6	+	+(0.5116)	−(0.428664)	+(0.919505)	38139.16	9.11	−(47.96)	84.16	−0.521
V7	+	+(0.5295)	+(0.702073)	+(0.980207)	26647.77	10.40	−(43.35)	71.88	−0.632
V8	+	+(0.4751)	+(0.717110)	+(0.859654)	34998.14	9.31	+(33.79)	85.38	−0.290
V9	+	+(0.4976)	+(0.500000)	+(0.850564)	39115.22	9.35	−(42.92)	81.13	−0.562
V10	+	+(0.4999)	−(0.420452)	+(0.919082)	38139.16	9.11	−(47.43)	84.16	−0.521
V11	+	+(0.5124)	+(0.670082)	+(0.978970)	26647.77	10.4	−(42.59)	71.88	−0.632
V12	+	+(0.4626)	+(0.721238)	+(0.846745)	34998.14	9.31	+(33.22)	85.38	−0.290
V13	+	+(0.4928)	+(0.500000)	+(0.863467)	39115.22	9.35	−(44.17)	81.13	−0.562
V14	+	+(0.4950)	−(0.406867)	+(0.917314)	38139.16	9.11	−(48.70)	84.16	−0.521
V15	+	+(0.5053)	+(0.668008)	+(0.979064)	26647.77	10.40	−(44.43)	71.88	−0.632
V16	+	+(0.4574)	+(0.686732)	+(0.850063)	34998.14	9.31	+(34.57)	85.38	−0.290

**Table 7:** Structural quality comparison of the modelled vaccine constructs.

Properties	V4	V8	V12	V16
** Quality factor **	99.05	94.32	95.87	95.06
** Ramachandran favored **	96.06%	93.5%	95.05%	92.88%
** Verify3D structural violations **	Pass	Pass	Fail	Pass
** Distribution Z-score **	−0.11 ± 0.43	−0.16 ± 0.43	1.47 ± 0.45	−0.35 ± 0.45
** Backbone dynamics (Average S^2^-parameter scores) **	0.79	0.81	0.81	0.81

**Table 8:** The relative binding parameter values of the docked complexes.

Vaccine Construct	Target (PDB IDs)	Global Energy (kcal/mol)	Attractive vdW	Atomic Contact Energy (kcal/mol)	Hydrogen Bond Contribution (kcal/mol)
Vaccine Construct-4 (V4)	1H15	−23.62	−42.33	14.93	−5.68
	2FSE	−17.38	−34.59	12.27	−3.15
	2Z65	−1.98	−2.30	2.60	0.00
	2Q6W	−0.97	−6.92	−0.01	−0.76
	2SEB	−0.61	−25.23	4.33	−1.63
	1A6A	1.45	−35.06	6.69	−2.41
	3C5J	2.74	−21.38	9.68	−4.05

## Data Availability

The data associated with the article have been included in the manuscript and/or its Supplementary Files.

## Supplementary Materials

The following supporting information can be downloaded at: https://www.mdpi.com/article/10.3390/v14112504/s1, Table S1: Backbone dynamics of the Constructs.
