# Supplementary material for: Multi-Epitope Vaccine for Monkeypox Using Pan-Genome and Reverse Vaccinology Approaches
Source: Viruses. 2022 Nov 12;14(11):2504. doi: 10.3390/v14112504 (PMC9695528; doi:10.3390/v14112504)
Supplement: Supplementary file 1 [file viruses-14-02504-s001.zip › viruses-2026717-supplementary.pdf]

**Supplementary File 1**

**Backbone dynamics of the Constructs**

| Residues | V4 | V8 | V12 | V16 |
|---|---|---|---|---|
| 1 | 0.764 | 0.764 | 0.764 | 0.764 |
| 2 | 0.7601 | 0.7601 | 0.7601 | 0.7601 |
| 3 | 0.7704 | 0.7704 | 0.7704 | 0.7704 |
| 4 | 0.7691 | 0.7691 | 0.7691 | 0.7691 |
| 5 | 0.7727 | 0.7727 | 0.7727 | 0.7727 |
| 6 | 0.7768 | 0.7768 | 0.7768 | 0.7768 |
| 7 | 0.7798 | 0.7798 | 0.7798 | 0.7798 |
| 8 | 0.7776 | 0.7776 | 0.7776 | 0.7776 |
| 9 | 0.79025 | 0.79025 | 0.79025 | 0.79025 |
| 10 | 0.81385 | 0.81385 | 0.81385 | 0.81385 |
| 11 | 0.8363 | 0.8363 | 0.8363 | 0.8363 |
| 12 | 0.83445 | 0.83445 | 0.83445 | 0.83445 |
| 13 | 0.84645 | 0.84645 | 0.84645 | 0.84645 |
| 14 | 0.8594 | 0.8594 | 0.8594 | 0.8594 |
| 15 | 0.88705 | 0.88705 | 0.88705 | 0.88705 |
| 16 | 0.89295 | 0.89295 | 0.89295 | 0.89295 |
| 17 | 0.89705 | 0.89705 | 0.89705 | 0.89705 |
| 18 | 0.8951 | 0.8951 | 0.8951 | 0.8951 |
| 19 | 0.8983 | 0.8983 | 0.8983 | 0.8983 |
| 20 | 0.8976 | 0.8976 | 0.8976 | 0.8976 |
| 21 | 0.89465 | 0.89465 | 0.89465 | 0.89465 |
| 22 | 0.8953 | 0.8953 | 0.8953 | 0.8953 |
| 23 | 0.8981 | 0.8981 | 0.8981 | 0.8981 |
| 24 | 0.8964 | 0.8964 | 0.8964 | 0.8964 |
| 25 | 0.9011 | 0.9011 | 0.9011 | 0.9011 |
| 26 | 0.9089 | 0.9089 | 0.9089 | 0.9089 |
| 27 | 0.9188 | 0.9188 | 0.9188 | 0.9188 |
| 28 | 0.9063 | 0.9063 | 0.9063 | 0.9063 |
| 29 | 0.9006 | 0.9006 | 0.9006 | 0.9006 |
| 30 | 0.8853 | 0.8853 | 0.8853 | 0.8853 |
| 31 | 0.8837 | 0.8837 | 0.8837 | 0.8837 |

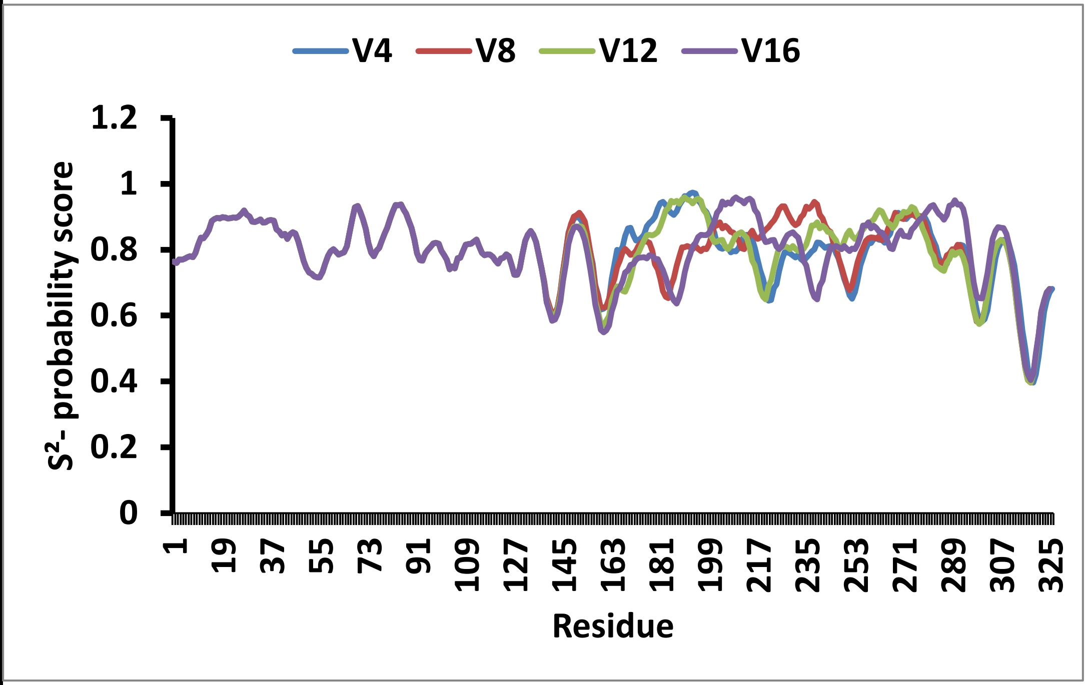


**Dynamics of Epitope-containing Domain**

| Residues | V4 | V8 | V12 | V16 |
|---|---|---|---|---|
| 131 | 0.8264 | 0.8264 | 0.8264 | 0.8264 |
| 132 | 0.8455 | 0.8455 | 0.8455 | 0.8455 |
| 133 | 0.8571 | 0.8571 | 0.8571 | 0.8571 |
| 134 | 0.8449 | 0.8449 | 0.8449 | 0.8449 |
| 135 | 0.8232 | 0.8232 | 0.8232 | 0.8232 |
| 136 | 0.7862 | 0.7862 | 0.7862 | 0.7862 |
| 137 | 0.7444 | 0.7444 | 0.7444 | 0.7444 |
| 138 | 0.6996 | 0.7066 | 0.6963 | 0.7001 |
| 139 | 0.6407 | 0.6544 | 0.645 | 0.6407 |
| 140 | 0.6192 | 0.6279 | 0.6156 | 0.6115 |
| 141 | 0.5972 | 0.6038 | 0.595 | 0.5843 |
| 142 | 0.6024 | 0.6072 | 0.6024 | 0.5877 |

| | | | | |
|---|---|---|---|---|
| 32 | 0.887 | 0.887 | 0.887 | 0.887 |
| 33 | 0.8923 | 0.8923 | 0.8923 | 0.8923 |
| 34 | 0.8822 | 0.8822 | 0.8822 | 0.8822 |
| 35 | 0.8834 | 0.8834 | 0.8834 | 0.8834 |
| 36 | 0.8894 | 0.8894 | 0.8894 | 0.8894 |
| 37 | 0.8898 | 0.8898 | 0.8898 | 0.8898 |
| 38 | 0.8876 | 0.8876 | 0.8876 | 0.8876 |
| 39 | 0.8619 | 0.8619 | 0.8619 | 0.8619 |
| 40 | 0.8552 | 0.8552 | 0.8552 | 0.8552 |
| 41 | 0.8431 | 0.8431 | 0.8431 | 0.8431 |
| 42 | 0.8482 | 0.8482 | 0.8482 | 0.8482 |
| 43 | 0.8331 | 0.8331 | 0.8331 | 0.8331 |
| 44 | 0.8463 | 0.8463 | 0.8463 | 0.8463 |
| 45 | 0.853 | 0.853 | 0.853 | 0.853 |
| 46 | 0.8487 | 0.8487 | 0.8487 | 0.8487 |
| 47 | 0.8261 | 0.8261 | 0.8261 | 0.8261 |
| 48 | 0.798 | 0.798 | 0.798 | 0.798 |
| 49 | 0.7654 | 0.7654 | 0.7654 | 0.7654 |
| 50 | 0.7453 | 0.7453 | 0.7453 | 0.7453 |
| 51 | 0.7308 | 0.7308 | 0.7308 | 0.7308 |
| 52 | 0.727 | 0.727 | 0.727 | 0.727 |
| 53 | 0.7194 | 0.7194 | 0.7194 | 0.7194 |
| 54 | 0.7163 | 0.7163 | 0.7163 | 0.7163 |
| 55 | 0.7157 | 0.7157 | 0.7157 | 0.7157 |
| 56 | 0.7322 | 0.7322 | 0.7322 | 0.7322 |
| 57 | 0.7572 | 0.7572 | 0.7572 | 0.7572 |
| 58 | 0.7807 | 0.7807 | 0.7807 | 0.7807 |
| 59 | 0.7944 | 0.7944 | 0.7944 | 0.7944 |
| 60 | 0.8019 | 0.8019 | 0.8019 | 0.8019 |
| 61 | 0.794 | 0.794 | 0.794 | 0.794 |
| 62 | 0.785 | 0.785 | 0.785 | 0.785 |
| 63 | 0.789 | 0.789 | 0.789 | 0.789 |
| 64 | 0.7916 | 0.7916 | 0.7916 | 0.7916 |
| 65 | 0.8105 | 0.8105 | 0.8105 | 0.8105 |

| | | | | |
|---|---|---|---|---|
| 143 | 0.6237 | 0.6251 | 0.618 | 0.6054 |
| 144 | 0.6721 | 0.6735 | 0.6588 | 0.6436 |
| 145 | 0.7346 | 0.7352 | 0.7185 | 0.704 |
| 146 | 0.796 | 0.7932 | 0.7718 | 0.7571 |
| 147 | 0.8521 | 0.8486 | 0.8216 | 0.8169 |
| 148 | 0.8744 | 0.8784 | 0.8536 | 0.8454 |
| 149 | 0.8936 | 0.901 | 0.8704 | 0.8665 |
| 150 | 0.8997 | 0.9047 | 0.8685 | 0.8703 |
| 151 | 0.8967 | 0.9126 | 0.8711 | 0.8653 |
| 152 | 0.8862 | 0.9016 | 0.8691 | 0.8543 |
| 153 | 0.8604 | 0.8859 | 0.8477 | 0.8285 |
| 154 | 0.8266 | 0.8476 | 0.81 | 0.7919 |
| 155 | 0.7934 | 0.7944 | 0.7663 | 0.742 |
| 156 | 0.7574 | 0.7459 | 0.7129 | 0.6916 |
| 157 | 0.6929 | 0.688 | 0.6444 | 0.6324 |
| 158 | 0.6612 | 0.6556 | 0.6109 | 0.5946 |
| 159 | 0.6177 | 0.6237 | 0.5742 | 0.5563 |
| 160 | 0.6204 | 0.6243 | 0.5699 | 0.5489 |
| 161 | 0.6272 | 0.6269 | 0.5839 | 0.5548 |
| 162 | 0.65 | 0.6516 | 0.5984 | 0.5702 |
| 163 | 0.7116 | 0.6872 | 0.6457 | 0.6122 |
| 164 | 0.7579 | 0.7124 | 0.6703 | 0.6388 |
| 165 | 0.7994 | 0.7473 | 0.6888 | 0.6751 |
| 166 | 0.7952 | 0.766 | 0.6834 | 0.6833 |
| 167 | 0.8082 | 0.7918 | 0.6755 | 0.7033 |
| 168 | 0.8416 | 0.8023 | 0.6725 | 0.7304 |
| 169 | 0.8642 | 0.7933 | 0.693 | 0.737 |
| 170 | 0.8665 | 0.7843 | 0.7161 | 0.754 |
| 171 | 0.8432 | 0.7879 | 0.7547 | 0.7571 |
| 172 | 0.8274 | 0.7965 | 0.7789 | 0.7694 |
| 173 | 0.8289 | 0.8123 | 0.7922 | 0.7753 |
| 174 | 0.8362 | 0.8216 | 0.8114 | 0.776 |
| 175 | 0.8448 | 0.8248 | 0.8275 | 0.7769 |
| 176 | 0.8717 | 0.8247 | 0.8445 | 0.7732 |

| | | | | |
|---|---|---|---|---|
| 66 | 0.8497 | 0.8497 | 0.8497 | 0.8497 |
| 67 | 0.8937 | 0.8937 | 0.8937 | 0.8937 |
| 68 | 0.9291 | 0.9291 | 0.9291 | 0.9291 |
| 69 | 0.9327 | 0.9327 | 0.9327 | 0.9327 |
| 70 | 0.9141 | 0.9141 | 0.9141 | 0.9141 |
| 71 | 0.8933 | 0.8933 | 0.8933 | 0.8933 |
| 72 | 0.863 | 0.863 | 0.863 | 0.863 |
| 73 | 0.8207 | 0.8207 | 0.8207 | 0.8207 |
| 74 | 0.7911 | 0.7911 | 0.7911 | 0.7911 |
| 75 | 0.78 | 0.78 | 0.78 | 0.78 |
| 76 | 0.7972 | 0.7972 | 0.7972 | 0.7972 |
| 77 | 0.805 | 0.805 | 0.805 | 0.805 |
| 78 | 0.8271 | 0.8271 | 0.8271 | 0.8271 |
| 79 | 0.8488 | 0.8488 | 0.8488 | 0.8488 |
| 80 | 0.8662 | 0.8662 | 0.8662 | 0.8662 |
| 81 | 0.8941 | 0.8941 | 0.8941 | 0.8941 |
| 82 | 0.9153 | 0.9153 | 0.9153 | 0.9153 |
| 83 | 0.9352 | 0.9352 | 0.9352 | 0.9352 |
| 84 | 0.9348 | 0.9348 | 0.9348 | 0.9348 |
| 85 | 0.9376 | 0.9376 | 0.9376 | 0.9376 |
| 86 | 0.9218 | 0.9218 | 0.9218 | 0.9218 |
| 87 | 0.9098 | 0.9098 | 0.9098 | 0.9098 |
| 88 | 0.8864 | 0.8864 | 0.8864 | 0.8864 |
| 89 | 0.8653 | 0.8653 | 0.8653 | 0.8653 |
| 90 | 0.8295 | 0.8295 | 0.8295 | 0.8295 |
| 91 | 0.7891 | 0.7891 | 0.7891 | 0.7891 |
| 92 | 0.7687 | 0.7687 | 0.7687 | 0.7687 |
| 93 | 0.7669 | 0.7669 | 0.7669 | 0.7669 |
| 94 | 0.7889 | 0.7889 | 0.7889 | 0.7889 |
| 95 | 0.7994 | 0.7994 | 0.7994 | 0.7994 |
| 96 | 0.808 | 0.808 | 0.808 | 0.808 |
| 97 | 0.8186 | 0.8186 | 0.8186 | 0.8186 |
| 98 | 0.8208 | 0.8208 | 0.8208 | 0.8208 |
| 99 | 0.818 | 0.818 | 0.818 | 0.818 |

| | | | | |
|---|---|---|---|---|
| 177 | 0.8808 | 0.8205 | 0.845 | 0.7834 |
| 178 | 0.8888 | 0.7964 | 0.8431 | 0.7808 |
| 179 | 0.8994 | 0.7554 | 0.847 | 0.7726 |
| 180 | 0.9246 | 0.7405 | 0.8543 | 0.7712 |
| 181 | 0.9411 | 0.7115 | 0.8706 | 0.7535 |
| 182 | 0.9452 | 0.6742 | 0.8925 | 0.7379 |
| 183 | 0.9357 | 0.657 | 0.9199 | 0.7141 |
| 184 | 0.9214 | 0.6529 | 0.9262 | 0.6864 |
| 185 | 0.911 | 0.6915 | 0.9484 | 0.6639 |
| 186 | 0.9057 | 0.7129 | 0.9438 | 0.646 |
| 187 | 0.9162 | 0.7507 | 0.9485 | 0.6362 |
| 188 | 0.9344 | 0.7783 | 0.9399 | 0.6549 |
| 189 | 0.9483 | 0.8075 | 0.9533 | 0.6865 |
| 190 | 0.9628 | 0.8077 | 0.9589 | 0.7298 |
| 191 | 0.9617 | 0.8112 | 0.9523 | 0.7611 |
| 192 | 0.9701 | 0.8074 | 0.9492 | 0.7851 |
| 193 | 0.9732 | 0.8017 | 0.9408 | 0.81 |
| 194 | 0.9707 | 0.8108 | 0.951 | 0.8206 |
| 195 | 0.9505 | 0.8026 | 0.9547 | 0.8384 |
| 196 | 0.9393 | 0.7959 | 0.948 | 0.8448 |
| 197 | 0.9226 | 0.803 | 0.9208 | 0.8438 |
| 198 | 0.9143 | 0.8012 | 0.9097 | 0.844 |
| 199 | 0.8973 | 0.8161 | 0.8809 | 0.8503 |
| 200 | 0.8754 | 0.8405 | 0.8388 | 0.8666 |
| 201 | 0.851 | 0.8734 | 0.8228 | 0.8849 |
| 202 | 0.8167 | 0.8734 | 0.8246 | 0.9125 |
| 203 | 0.8059 | 0.8835 | 0.8284 | 0.9227 |
| 204 | 0.8022 | 0.8642 | 0.8296 | 0.9464 |
| 205 | 0.8075 | 0.8724 | 0.8092 | 0.9369 |
| 206 | 0.807 | 0.8657 | 0.8019 | 0.9433 |
| 207 | 0.7921 | 0.8537 | 0.811 | 0.9399 |
| 208 | 0.7957 | 0.852 | 0.8289 | 0.9533 |
| 209 | 0.795 | 0.8394 | 0.844 | 0.9589 |
| 210 | 0.8055 | 0.8279 | 0.8492 | 0.9523 |

| | | | | |
|---|---|---|---|---|
| 100 | 0.7963 | 0.7963 | 0.7963 | 0.7963 |
| 101 | 0.7835 | 0.7835 | 0.7835 | 0.7835 |
| 102 | 0.7699 | 0.7699 | 0.7699 | 0.7699 |
| 103 | 0.7402 | 0.7402 | 0.7402 | 0.7402 |
| 104 | 0.7488 | 0.7488 | 0.7488 | 0.7488 |
| 105 | 0.7434 | 0.7434 | 0.7434 | 0.7434 |
| 106 | 0.7743 | 0.7743 | 0.7743 | 0.7743 |
| 107 | 0.7751 | 0.7751 | 0.7751 | 0.7751 |
| 108 | 0.7969 | 0.7969 | 0.7969 | 0.7969 |
| 109 | 0.8156 | 0.8156 | 0.8156 | 0.8156 |
| 110 | 0.8174 | 0.8174 | 0.8174 | 0.8174 |
| 111 | 0.8184 | 0.8184 | 0.8184 | 0.8184 |
| 112 | 0.8248 | 0.8248 | 0.8248 | 0.8248 |
| 113 | 0.8302 | 0.8302 | 0.8302 | 0.8302 |
| 114 | 0.8103 | 0.8103 | 0.8103 | 0.8103 |
| 115 | 0.7903 | 0.7903 | 0.7903 | 0.7903 |
| 116 | 0.7826 | 0.7826 | 0.7826 | 0.7826 |
| 117 | 0.7856 | 0.7856 | 0.7856 | 0.7856 |
| 118 | 0.7847 | 0.7847 | 0.7847 | 0.7847 |
| 119 | 0.778 | 0.778 | 0.778 | 0.778 |
| 120 | 0.7664 | 0.7664 | 0.7664 | 0.7664 |
| 121 | 0.7578 | 0.7578 | 0.7578 | 0.7578 |
| 122 | 0.7734 | 0.7734 | 0.7734 | 0.7734 |
| 123 | 0.7751 | 0.7751 | 0.7751 | 0.7751 |
| 124 | 0.7859 | 0.7859 | 0.7859 | 0.7859 |
| 125 | 0.7797 | 0.7797 | 0.7797 | 0.7797 |
| 126 | 0.752 | 0.752 | 0.752 | 0.752 |
| 127 | 0.724 | 0.724 | 0.724 | 0.724 |
| 128 | 0.724 | 0.724 | 0.724 | 0.724 |
| 129 | 0.7432 | 0.7432 | 0.7432 | 0.7432 |
| 130 | 0.7847 | 0.7847 | 0.7847 | 0.7847 |
| 131 | 0.8264 | 0.8264 | 0.8264 | 0.8264 |
| 132 | 0.8455 | 0.8455 | 0.8455 | 0.8455 |
| 133 | 0.8571 | 0.8571 | 0.8571 | 0.8571 |

| | | | | |
|---|---|---|---|---|
| 211 | 0.8252 | 0.8041 | 0.8529 | 0.9492 |
| 212 | 0.8435 | 0.8019 | 0.844 | 0.9408 |
| 213 | 0.8454 | 0.825 | 0.8362 | 0.951 |
| 214 | 0.8338 | 0.8476 | 0.8064 | 0.9547 |
| 215 | 0.8316 | 0.8579 | 0.7669 | 0.948 |
| 216 | 0.8065 | 0.8415 | 0.7523 | 0.9208 |
| 217 | 0.7696 | 0.8328 | 0.7162 | 0.9097 |
| 218 | 0.7378 | 0.8365 | 0.677 | 0.8809 |
| 219 | 0.7108 | 0.852 | 0.6569 | 0.8388 |
| 220 | 0.6748 | 0.861 | 0.6494 | 0.8228 |
| 221 | 0.6454 | 0.8689 | 0.6895 | 0.8246 |
| 222 | 0.6473 | 0.879 | 0.706 | 0.8284 |
| 223 | 0.684 | 0.8888 | 0.7455 | 0.8296 |
| 224 | 0.6947 | 0.9028 | 0.7783 | 0.8092 |
| 225 | 0.7344 | 0.9218 | 0.8075 | 0.8019 |
| 226 | 0.7692 | 0.9322 | 0.8077 | 0.811 |
| 227 | 0.7922 | 0.9314 | 0.8112 | 0.8289 |
| 228 | 0.7919 | 0.9123 | 0.8074 | 0.844 |
| 229 | 0.7888 | 0.8965 | 0.8017 | 0.8492 |
| 230 | 0.7824 | 0.882 | 0.8108 | 0.8529 |
| 231 | 0.7765 | 0.874 | 0.8026 | 0.844 |
| 232 | 0.7851 | 0.8804 | 0.7959 | 0.8362 |
| 233 | 0.7711 | 0.8982 | 0.803 | 0.8064 |
| 234 | 0.7651 | 0.9068 | 0.8012 | 0.7669 |
| 235 | 0.7756 | 0.9303 | 0.8161 | 0.7523 |
| 236 | 0.7836 | 0.9235 | 0.8405 | 0.7162 |
| 237 | 0.7952 | 0.9325 | 0.8734 | 0.677 |
| 238 | 0.8008 | 0.9451 | 0.8734 | 0.6569 |
| 239 | 0.8224 | 0.9377 | 0.8835 | 0.6494 |
| 240 | 0.8207 | 0.9069 | 0.8642 | 0.6895 |
| 241 | 0.8136 | 0.8946 | 0.8724 | 0.706 |
| 242 | 0.8056 | 0.8731 | 0.8657 | 0.7455 |
| 243 | 0.8091 | 0.8599 | 0.8537 | 0.7783 |
| 244 | 0.8108 | 0.8543 | 0.852 | 0.8075 |

| | | | | |
|---|---|---|---|---|
| 134 | 0.8449 | 0.8449 | 0.8449 | 0.8449 |
| 135 | 0.8232 | 0.8232 | 0.8232 | 0.8232 |
| 136 | 0.7862 | 0.7862 | 0.7862 | 0.7862 |
| 137 | 0.7444 | 0.7444 | 0.7444 | 0.7444 |
| 138 | 0.6996 | 0.7066 | 0.6963 | 0.7001 |
| 139 | 0.6407 | 0.6544 | 0.645 | 0.6407 |
| 140 | 0.6192 | 0.6279 | 0.6156 | 0.6115 |
| 141 | 0.5972 | 0.6038 | 0.595 | 0.5843 |
| 142 | 0.6024 | 0.6072 | 0.6024 | 0.5877 |
| 143 | 0.6237 | 0.6251 | 0.618 | 0.6054 |
| 144 | 0.6721 | 0.6735 | 0.6588 | 0.6436 |
| 145 | 0.7346 | 0.7352 | 0.7185 | 0.704 |
| 146 | 0.796 | 0.7932 | 0.7718 | 0.7571 |
| 147 | 0.8521 | 0.8486 | 0.8216 | 0.8169 |
| 148 | 0.8744 | 0.8784 | 0.8536 | 0.8454 |
| 149 | 0.8936 | 0.901 | 0.8704 | 0.8665 |
| 150 | 0.8997 | 0.9047 | 0.8685 | 0.8703 |
| 151 | 0.8967 | 0.9126 | 0.8711 | 0.8653 |
| 152 | 0.8862 | 0.9016 | 0.8691 | 0.8543 |
| 153 | 0.8604 | 0.8859 | 0.8477 | 0.8285 |
| 154 | 0.8266 | 0.8476 | 0.81 | 0.7919 |
| 155 | 0.7934 | 0.7944 | 0.7663 | 0.742 |
| 156 | 0.7574 | 0.7459 | 0.7129 | 0.6916 |
| 157 | 0.6929 | 0.688 | 0.6444 | 0.6324 |
| 158 | 0.6612 | 0.6556 | 0.6109 | 0.5946 |
| 159 | 0.6177 | 0.6237 | 0.5742 | 0.5563 |
| 160 | 0.6204 | 0.6243 | 0.5699 | 0.5489 |
| 161 | 0.6272 | 0.6269 | 0.5839 | 0.5548 |
| 162 | 0.65 | 0.6516 | 0.5984 | 0.5702 |
| 163 | 0.7116 | 0.6872 | 0.6457 | 0.6122 |
| 164 | 0.7579 | 0.7124 | 0.6703 | 0.6388 |
| 165 | 0.7994 | 0.7473 | 0.6888 | 0.6751 |
| 166 | 0.7952 | 0.766 | 0.6834 | 0.6833 |
| 167 | 0.8082 | 0.7918 | 0.6755 | 0.7033 |

| | | | | |
|---|---|---|---|---|
| 245 | 0.8142 | 0.8222 | 0.8394 | 0.8077 |
| 246 | 0.7893 | 0.8095 | 0.8279 | 0.8112 |
| 247 | 0.7728 | 0.7746 | 0.8041 | 0.8074 |
| 248 | 0.7416 | 0.7474 | 0.8019 | 0.8017 |
| 249 | 0.7118 | 0.7196 | 0.825 | 0.8108 |
| 250 | 0.6876 | 0.6912 | 0.8476 | 0.8026 |
| 251 | 0.6618 | 0.6772 | 0.8579 | 0.7959 |
| 252 | 0.6513 | 0.6918 | 0.8415 | 0.803 |
| 253 | 0.6718 | 0.7275 | 0.8328 | 0.8012 |
| 254 | 0.7049 | 0.7618 | 0.8365 | 0.8161 |
| 255 | 0.752 | 0.7879 | 0.852 | 0.8405 |
| 256 | 0.7756 | 0.8051 | 0.861 | 0.8734 |
| 257 | 0.8019 | 0.8268 | 0.8727 | 0.8734 |
| 258 | 0.8202 | 0.8344 | 0.8747 | 0.8835 |
| 259 | 0.8204 | 0.8372 | 0.8847 | 0.8642 |
| 260 | 0.8331 | 0.8366 | 0.8921 | 0.8724 |
| 261 | 0.8345 | 0.8337 | 0.9071 | 0.8657 |
| 262 | 0.8294 | 0.8415 | 0.9196 | 0.8537 |
| 263 | 0.8296 | 0.8289 | 0.9162 | 0.852 |
| 264 | 0.821 | 0.8376 | 0.8978 | 0.8394 |
| 265 | 0.8361 | 0.8573 | 0.8818 | 0.8279 |
| 266 | 0.8477 | 0.8815 | 0.8773 | 0.8041 |
| 267 | 0.8795 | 0.8897 | 0.8658 | 0.8019 |
| 268 | 0.8897 | 0.9119 | 0.8765 | 0.825 |
| 269 | 0.9119 | 0.9025 | 0.9 | 0.8476 |
| 270 | 0.9025 | 0.896 | 0.901 | 0.8579 |
| 271 | 0.896 | 0.8926 | 0.9155 | 0.8415 |
| 272 | 0.8926 | 0.904 | 0.9124 | 0.8409 |
| 273 | 0.904 | 0.9051 | 0.9163 | 0.8384 |
| 274 | 0.9051 | 0.9088 | 0.9297 | 0.8609 |
| 275 | 0.9088 | 0.9036 | 0.9251 | 0.8697 |
| 276 | 0.9036 | 0.8977 | 0.9074 | 0.8814 |
| 277 | 0.8977 | 0.9024 | 0.8907 | 0.8914 |
| 278 | 0.9024 | 0.8964 | 0.8643 | 0.8979 |

| | | | | |
|---|---|---|---|---|
| 168 | 0.8416 | 0.8023 | 0.6725 | 0.7304 |
| 169 | 0.8642 | 0.7933 | 0.693 | 0.737 |
| 170 | 0.8665 | 0.7843 | 0.7161 | 0.754 |
| 171 | 0.8432 | 0.7879 | 0.7547 | 0.7571 |
| 172 | 0.8274 | 0.7965 | 0.7789 | 0.7694 |
| 173 | 0.8289 | 0.8123 | 0.7922 | 0.7753 |
| 174 | 0.8362 | 0.8216 | 0.8114 | 0.776 |
| 175 | 0.8448 | 0.8248 | 0.8275 | 0.7769 |
| 176 | 0.8717 | 0.8247 | 0.8445 | 0.7732 |
| 177 | 0.8808 | 0.8205 | 0.845 | 0.7834 |
| 178 | 0.8888 | 0.7964 | 0.8431 | 0.7808 |
| 179 | 0.8994 | 0.7554 | 0.847 | 0.7726 |
| 180 | 0.9246 | 0.7405 | 0.8543 | 0.7712 |
| 181 | 0.9411 | 0.7115 | 0.8706 | 0.7535 |
| 182 | 0.9452 | 0.6742 | 0.8925 | 0.7379 |
| 183 | 0.9357 | 0.657 | 0.9199 | 0.7141 |
| 184 | 0.9214 | 0.6529 | 0.9262 | 0.6864 |
| 185 | 0.911 | 0.6915 | 0.9484 | 0.6639 |
| 186 | 0.9057 | 0.7129 | 0.9438 | 0.646 |
| 187 | 0.9162 | 0.7507 | 0.9485 | 0.6362 |
| 188 | 0.9344 | 0.7783 | 0.9399 | 0.6549 |
| 189 | 0.9483 | 0.8075 | 0.9533 | 0.6865 |
| 190 | 0.9628 | 0.8077 | 0.9589 | 0.7298 |
| 191 | 0.9617 | 0.8112 | 0.9523 | 0.7611 |
| 192 | 0.9701 | 0.8074 | 0.9492 | 0.7851 |
| 193 | 0.9732 | 0.8017 | 0.9408 | 0.81 |
| 194 | 0.9707 | 0.8108 | 0.951 | 0.8206 |
| 195 | 0.9505 | 0.8026 | 0.9547 | 0.8384 |
| 196 | 0.9393 | 0.7959 | 0.948 | 0.8448 |
| 197 | 0.9226 | 0.803 | 0.9208 | 0.8438 |
| 198 | 0.9143 | 0.8012 | 0.9097 | 0.844 |
| 199 | 0.8973 | 0.8161 | 0.8809 | 0.8503 |
| 200 | 0.8754 | 0.8405 | 0.8388 | 0.8666 |
| 201 | 0.851 | 0.8734 | 0.8228 | 0.8849 |

| | | | | |
|---|---|---|---|---|
| 279 | 0.8964 | 0.8795 | 0.8458 | 0.9097 |
| 280 | 0.8795 | 0.8478 | 0.8243 | 0.9209 |
| 281 | 0.8478 | 0.8281 | 0.7938 | 0.932 |
| 282 | 0.8281 | 0.8098 | 0.7799 | 0.9353 |
| 283 | 0.8098 | 0.7746 | 0.7525 | 0.9217 |
| 284 | 0.7746 | 0.7626 | 0.7457 | 0.9085 |
| 285 | 0.7626 | 0.7615 | 0.7391 | 0.9016 |
| 286 | 0.7615 | 0.7567 | 0.7358 | 0.8908 |
| 287 | 0.7567 | 0.7832 | 0.7606 | 0.9059 |
| 288 | 0.7832 | 0.7882 | 0.7711 | 0.9335 |
| 289 | 0.7882 | 0.8014 | 0.7952 | 0.9366 |
| 290 | 0.8014 | 0.7999 | 0.7839 | 0.95 |
| 291 | 0.7999 | 0.8151 | 0.7917 | 0.9368 |
| 292 | 0.8151 | 0.8123 | 0.7935 | 0.9378 |
| 293 | 0.8123 | 0.7824 | 0.7767 | 0.9239 |
| 294 | 0.7824 | 0.7584 | 0.7509 | 0.8911 |
| 295 | 0.7584 | 0.7174 | 0.7072 | 0.8318 |
| 296 | 0.7174 | 0.6637 | 0.6591 | 0.7592 |
| 297 | 0.6637 | 0.6185 | 0.6186 | 0.7008 |
| 298 | 0.6185 | 0.5819 | 0.5846 | 0.6577 |
| 299 | 0.5819 | 0.5815 | 0.5742 | 0.6528 |
| 300 | 0.5815 | 0.5883 | 0.5818 | 0.6523 |
| 301 | 0.5883 | 0.61575 | 0.61385 | 0.68455 |
| 302 | 0.61575 | 0.66935 | 0.66275 | 0.72995 |
| 303 | 0.66935 | 0.7186 | 0.7186 | 0.7831 |
| 304 | 0.7186 | 0.7747 | 0.7747 | 0.8324 |
| 305 | 0.7747 | 0.8044 | 0.8044 | 0.8565 |
| 306 | 0.8044 | 0.8224 | 0.8224 | 0.8686 |
| 307 | 0.8224 | 0.83 | 0.83 | 0.8669 |
| 308 | 0.83 | 0.8249 | 0.8249 | 0.8659 |
| 309 | 0.8249 | 0.8157 | 0.8157 | 0.8477 |
| 310 | 0.8157 | 0.7891 | 0.7891 | 0.8159 |
| 311 | 0.7891 | 0.7518 | 0.7518 | 0.7718 |
| 312 | 0.7518 | 0.6951 | 0.6951 | 0.7184 |

| | | | | |
|---|---|---|---|---|
| 202 | 0.8167 | 0.8734 | 0.8246 | 0.9125 |
| 203 | 0.8059 | 0.8835 | 0.8284 | 0.9227 |
| 204 | 0.8022 | 0.8642 | 0.8296 | 0.9464 |
| 205 | 0.8075 | 0.8724 | 0.8092 | 0.9369 |
| 206 | 0.807 | 0.8657 | 0.8019 | 0.9433 |
| 207 | 0.7921 | 0.8537 | 0.811 | 0.9399 |
| 208 | 0.7957 | 0.852 | 0.8289 | 0.9533 |
| 209 | 0.795 | 0.8394 | 0.844 | 0.9589 |
| 210 | 0.8055 | 0.8279 | 0.8492 | 0.9523 |
| 211 | 0.8252 | 0.8041 | 0.8529 | 0.9492 |
| 212 | 0.8435 | 0.8019 | 0.844 | 0.9408 |
| 213 | 0.8454 | 0.825 | 0.8362 | 0.951 |
| 214 | 0.8338 | 0.8476 | 0.8064 | 0.9547 |
| 215 | 0.8316 | 0.8579 | 0.7669 | 0.948 |
| 216 | 0.8065 | 0.8415 | 0.7523 | 0.9208 |
| 217 | 0.7696 | 0.8328 | 0.7162 | 0.9097 |
| 218 | 0.7378 | 0.8365 | 0.677 | 0.8809 |
| 219 | 0.7108 | 0.852 | 0.6569 | 0.8388 |
| 220 | 0.6748 | 0.861 | 0.6494 | 0.8228 |
| 221 | 0.6454 | 0.8689 | 0.6895 | 0.8246 |
| 222 | 0.6473 | 0.879 | 0.706 | 0.8284 |
| 223 | 0.684 | 0.8888 | 0.7455 | 0.8296 |
| 224 | 0.6947 | 0.9028 | 0.7783 | 0.8092 |
| 225 | 0.7344 | 0.9218 | 0.8075 | 0.8019 |
| 226 | 0.7692 | 0.9322 | 0.8077 | 0.811 |
| 227 | 0.7922 | 0.9314 | 0.8112 | 0.8289 |
| 228 | 0.7919 | 0.9123 | 0.8074 | 0.844 |
| 229 | 0.7888 | 0.8965 | 0.8017 | 0.8492 |
| 230 | 0.7824 | 0.882 | 0.8108 | 0.8529 |
| 231 | 0.7765 | 0.874 | 0.8026 | 0.844 |
| 232 | 0.7851 | 0.8804 | 0.7959 | 0.8362 |
| 233 | 0.7711 | 0.8982 | 0.803 | 0.8064 |
| 234 | 0.7651 | 0.9068 | 0.8012 | 0.7669 |
| 235 | 0.7756 | 0.9303 | 0.8161 | 0.7523 |

| | | | | |
|---|---|---|---|---|
| 313 | 0.6951 | 0.62445 | 0.62445 | 0.65195 |
| 314 | 0.62445 | 0.5547 | 0.5547 | 0.5699 |
| 315 | 0.5547 | 0.5 | 0.5 | 0.5113 |
| 316 | 0.5 | 0.4395 | 0.4395 | 0.4489 |
| 317 | 0.4395 | 0.40435 | 0.40435 | 0.42155 |
| 318 | 0.40435 | 0.39595 | 0.39595 | 0.40445 |
| 319 | 0.39595 | 0.42075 | 0.42075 | 0.42295 |
| 320 | 0.42075 | 0.4775 | 0.4775 | 0.486 |
| 321 | 0.4775 | 0.54305 | 0.54305 | 0.54375 |
| 322 | 0.54305 | 0.61205 | 0.61205 | 0.61205 |
| 323 | 0.61205 | 0.6457 | 0.6457 | 0.6457 |
| 324 | 0.6457 | 0.6708 | 0.6708 | 0.6708 |
| 325 | 0.6708 | 0.6811 | 0.6811 | 0.6811 |
| 326 | 0.6811 | | | |
| **Average** | **0.78381** | **0.785029** | **0.785029** | **0.785029** |

| | | | | |
|---|---|---|---|---|
| 236 | 0.7836 | 0.9235 | 0.8405 | 0.7162 |
| 237 | 0.7952 | 0.9325 | 0.8734 | 0.677 |
| 238 | 0.8008 | 0.9451 | 0.8734 | 0.6569 |
| 239 | 0.8224 | 0.9377 | 0.8835 | 0.6494 |
| 240 | 0.8207 | 0.9069 | 0.8642 | 0.6895 |
| 241 | 0.8136 | 0.8946 | 0.8724 | 0.706 |
| 242 | 0.8056 | 0.8731 | 0.8657 | 0.7455 |
| 243 | 0.8091 | 0.8599 | 0.8537 | 0.7783 |
| 244 | 0.8108 | 0.8543 | 0.852 | 0.8075 |
| 245 | 0.8142 | 0.8222 | 0.8394 | 0.8077 |
| 246 | 0.7893 | 0.8095 | 0.8279 | 0.8112 |
| 247 | 0.7728 | 0.7746 | 0.8041 | 0.8074 |
| 248 | 0.7416 | 0.7474 | 0.8019 | 0.8017 |
| 249 | 0.7118 | 0.7196 | 0.825 | 0.8108 |
| 250 | 0.6876 | 0.6912 | 0.8476 | 0.8026 |
| 251 | 0.6618 | 0.6772 | 0.8579 | 0.7959 |
| 252 | 0.6513 | 0.6918 | 0.8415 | 0.803 |
| 253 | 0.6718 | 0.7275 | 0.8328 | 0.8012 |
| 254 | 0.7049 | 0.7618 | 0.8365 | 0.8161 |
| 255 | 0.752 | 0.7879 | 0.852 | 0.8405 |
| 256 | 0.7756 | 0.8051 | 0.861 | 0.8734 |
| 257 | 0.8019 | 0.8268 | 0.8727 | 0.8734 |
| 258 | 0.8202 | 0.8344 | 0.8747 | 0.8835 |
| 259 | 0.8204 | 0.8372 | 0.8847 | 0.8642 |
| 260 | 0.8331 | 0.8366 | 0.8921 | 0.8724 |
| 261 | 0.8345 | 0.8337 | 0.9071 | 0.8657 |
| 262 | 0.8294 | 0.8415 | 0.9196 | 0.8537 |
| 263 | 0.8296 | 0.8289 | 0.9162 | 0.852 |
| 264 | 0.821 | 0.8376 | 0.8978 | 0.8394 |
| 265 | 0.8361 | 0.8573 | 0.8818 | 0.8279 |
| 266 | 0.8477 | 0.8815 | 0.8773 | 0.8041 |
| 267 | 0.8795 | 0.8897 | 0.8658 | 0.8019 |
| 268 | 0.8897 | 0.9119 | 0.8765 | 0.825 |
| 269 | 0.9119 | 0.9025 | 0.9 | 0.8476 |

| | | | | |
|---|---|---|---|---|
| 270 | 0.9025 | 0.896 | 0.901 | 0.8579 |
| 271 | 0.896 | 0.8926 | 0.9155 | 0.8415 |
| 272 | 0.8926 | 0.904 | 0.9124 | 0.8409 |
| 273 | 0.904 | 0.9051 | 0.9163 | 0.8384 |
| 274 | 0.9051 | 0.9088 | 0.9297 | 0.8609 |
| 275 | 0.9088 | 0.9036 | 0.9251 | 0.8697 |
| 276 | 0.9036 | 0.8977 | 0.9074 | 0.8814 |
| 277 | 0.8977 | 0.9024 | 0.8907 | 0.8914 |
| 278 | 0.9024 | 0.8964 | 0.8643 | 0.8979 |
| 279 | 0.8964 | 0.8795 | 0.8458 | 0.9097 |
| 280 | 0.8795 | 0.8478 | 0.8243 | 0.9209 |
| 281 | 0.8478 | 0.8281 | 0.7938 | 0.932 |
| 282 | 0.8281 | 0.8098 | 0.7799 | 0.9353 |
| 283 | 0.8098 | 0.7746 | 0.7525 | 0.9217 |
| 284 | 0.7746 | 0.7626 | 0.7457 | 0.9085 |
| 285 | 0.7626 | 0.7615 | 0.7391 | 0.9016 |
| 286 | 0.7615 | 0.7567 | 0.7358 | 0.8908 |
| 287 | 0.7567 | 0.7832 | 0.7606 | 0.9059 |
| 288 | 0.7832 | 0.7882 | 0.7711 | 0.9335 |
| 289 | 0.7882 | 0.8014 | 0.7952 | 0.9366 |
| 290 | 0.8014 | 0.7999 | 0.7839 | 0.95 |
| 291 | 0.7999 | 0.8151 | 0.7917 | 0.9368 |
| 292 | 0.8151 | 0.8123 | 0.7935 | 0.9378 |
| 293 | 0.8123 | 0.7824 | 0.7767 | 0.9239 |
| 294 | 0.7824 | 0.7584 | 0.7509 | 0.8911 |
| 295 | 0.7584 | 0.7174 | 0.7072 | 0.8318 |
| 296 | 0.7174 | 0.6637 | 0.6591 | 0.7592 |
| 297 | 0.6637 | 0.6185 | 0.6186 | 0.7008 |
| 298 | 0.6185 | 0.5819 | 0.5846 | 0.6577 |
| 299 | 0.5819 | 0.5815 | 0.5742 | 0.6528 |
| 300 | 0.5815 | 0.5883 | 0.5818 | 0.6523 |
| 301 | 0.5883 | 0.61575 | 0.61385 | 0.68455 |
| 302 | 0.61575 | 0.66935 | 0.66275 | 0.72995 |
| 303 | 0.66935 | 0.7186 | 0.7186 | 0.7831 |

| | | | | |
|---|---|---|---|---|
| 304 | 0.7186 | 0.7747 | 0.7747 | 0.8324 |
| 305 | 0.7747 | 0.8044 | 0.8044 | 0.8565 |
| 306 | 0.8044 | 0.8224 | 0.8224 | 0.8686 |
| 307 | 0.8224 | 0.83 | 0.83 | 0.8669 |
| 308 | 0.83 | 0.8249 | 0.8249 | 0.8659 |
| 309 | 0.8249 | 0.8157 | 0.8157 | 0.8477 |
| 310 | 0.8157 | 0.7891 | 0.7891 | 0.8159 |
| 311 | 0.7891 | 0.7518 | 0.7518 | 0.7718 |
| 312 | 0.7518 | 0.6951 | 0.6951 | 0.7184 |
| 313 | 0.6951 | 0.62445 | 0.62445 | 0.65195 |
| 314 | 0.62445 | 0.5547 | 0.5547 | 0.5699 |
| 315 | 0.5547 | 0.5 | 0.5 | 0.5113 |
| 316 | 0.5 | 0.4395 | 0.4395 | 0.4489 |
| 317 | 0.4395 | 0.40435 | 0.40435 | 0.42155 |
| 318 | 0.40435 | 0.39595 | 0.39595 | 0.40445 |
| 319 | 0.39595 | 0.42075 | 0.42075 | 0.42295 |
| 320 | 0.42075 | 0.4775 | 0.4775 | 0.486 |
| 321 | 0.4775 | 0.54305 | 0.54305 | 0.54375 |
| 322 | 0.54305 | 0.61205 | 0.61205 | 0.61205 |
| 323 | 0.61205 | 0.6457 | 0.6457 | 0.6457 |
| 324 | 0.6457 | 0.6708 | 0.6708 | 0.6708 |
| 325 | 0.6708 | 0.6811 | 0.6811 | 0.6811 |
| 326 | 0.6811 | | | |
| **Average** | **0.799906** | **0.800686** | **0.800686** | **0.800686** |
